# Efficacy of different acupuncture therapies on postherpetic neuralgia: A Bayesian network meta-analysis

**DOI:** 10.3389/fnins.2022.1056102

**Published:** 2023-01-10

**Authors:** Yang Cui, Xinyu Zhou, Quan Li, Delong Wang, Jiamin Zhu, Xiangxin Zeng, Qichen Han, Rui Yang, Siyu Xu, Dongxu Zhang, Xiangyue Meng, Shuo Zhang, Zhongren Sun, Hongna Yin

**Affiliations:** ^1^Heilongjiang University of Chinese Medicine, Harbin, China; ^2^The Second Affiliated Hospital of Heilongjiang University of Chinese Medicine, Harbin, China; ^3^The First Affiliated Hospital of Heilongjiang University of Chinese Medicine, Harbin, China

**Keywords:** acupuncture, acupuncture analgesia, postherpetic neuralgia, systematic review, network meta-analysis

## Abstract

**Background:**

Postherpetic neuralgia (PHN) is a common, complex, and refractory type of neuropathic pain. Several systematic reviews support the efficacy of acupuncture and related treatments for PHN. Nevertheless, the efficacy of various acupuncture-related treatments for PHN remains debatable.

**Objective:**

We aimed to assess the efficacy and safety of acupuncture-related treatments for PHN, identify the most effective acupuncture-related treatments, and expound on the current inadequacies and prospects in the applications of acupuncture-related therapies.

**Methods:**

We searched PubMed, Cochrane Central Register of Controlled Trials, Embase, Web of Science, Google Scholar, four Chinese databases (China National Knowledge Infrastructure, China Biomedical, Chongqing VIP, and Wan Fang databases), clinical research registration platform (World Health Organization International Clinical Trial Registration platform, China Clinical Trial Registration Center) for relevant studies. We also examined previous meta-analyses; gray literature; and reference lists of the selected studies. We then evaluated the risk of bias in the included studies and performed a Bayesian multiple network meta-analysis.

**Results:**

We included 29 randomized controlled trials comprising 1,973 patients, of which five studies showed a high risk of bias. The pairwise meta-analysis results revealed that the efficacy of all acupuncture-related treatments for pain relief related to PHN was significantly better than antiepileptics. The network meta-analysis results showed that pricking and cupping plus antiepileptics were the most effective treatment, followed by electroacupuncture (EA) plus antiepileptics for pain relief in patients with PHN. EA plus antiepileptics ranked the best regarding reduced Pittsburgh Sleep Quality Index (PSQI) and Self-Rating Depression Scale (SDS) scores in patients with PHN. No results were found regarding the total response rate or quality of life in this study. Acupuncture-related treatments showed a lower incidence of adverse events than that of antiepileptics.

**Conclusion:**

Acupuncture-related therapies are potential treatment options for PHN and are safe. Pricking and cupping plus antiepileptics, are the most effective acupuncture-related techniques for pain relief, while EA plus antiepileptics is the best acupuncture-related technique for improving PHN-related insomnia and depression symptoms. However, owing to the limitations of this study, these conclusions should be cautiously interpreted, and future high-quality studies are needed.

**Systematic review registration:**

https://www.crd.york.ac.uk/prospero/display_record.php?ID=CRD42021226422, identifier CRD42021226422.

## 1. Introduction

Postherpetic neuralgia (PHN) is a complex and refractory type of neuropathy (Baron and Wasner, 2006), and is the most frequent chronic complication of herpes zoster (shingles) infection (Johnson and Rice, 2014). Although there are multiple definitions of PHN, the most conventionally used criterion is dermatomal pain persisting for at least 90 days after acute herpes zoster rash onset (Cohen, 2013; Scholz et al., 2019). The case definition of PHN in clinical trials often includes a minimum threshold of clinically significant pain intensity, which is usually a score of 40 or higher (sometimes ≥ 30) on a Likert scale of 0–100 (Oxman et al., 2005; Dworkin et al., 2010). Pain associated with PHN can be classified into three categories: persistent spontaneous pain (e.g., persistent burning pain), paroxysmal shooting or electric shock-like pain, evoked pain that includes allodynia (pain triggered from the innocuous stimulus), and hyperalgesia that may persist for several months or even years (Jensen and Finnerup, 2014; Finnerup et al., 2021).

The incidence and prevalence of PHN vary according to the definition used; 19.5 and 13.7% of patients with herpes zoster developed PHN at least 1 month and 3 months after symptom onset, respectively (Gauthier et al., 2009). One meta-analysis showed an annual incidence rate for PHN of 3.9–42.0 per 100,000 people (van Hecke et al., 2014). Like herpes zoster, a positive correlation has been seen between PHN severity or prevalence and age (Zin et al., 2008; Mallick-Searle et al., 2016), and the proportion of patients with spontaneous symptom remission decreases with age (Johnson and Rice, 2014). Other than older age, PHN risk factors may include the clinical features of acute herpes zoster infection [including prodromal pain, severe acute pain, severe skin lesions, and herpes rash in specific areas (e.g., ophthalmic symptoms)], chronic diseases, diabetes mellitus, and severe immunosuppression (Drolet et al., 2010; Forbes et al., 2016a,b; Wei et al., 2019). Family members of patients with PHN are also prone to fatigue, stress, insomnia, and emotional distress (Weinke et al., 2014). PHN causes considerable pain, resulting in a global healthcare burden for individuals and society (Doth et al., 2010; Friesen et al., 2016).

Unfortunately, PHN management remains challenging. The only well-documented means of PHN prevention is herpes zoster prevention (Johnson and Rice, 2014). However, once a patient develops PHN, the primary goal is symptom control, including pain reduction and emotional and sleep regulation. Existing evidence supports the use of oral drugs, including antiepileptics (e.g., pregabalin and gabapentin), tricyclic antidepressants (e.g., amitriptyline), or topical treatments (e.g., 5% lidocaine or capsaicin patches) as first-line treatments for PHN (Johnson and Rice, 2014; Moore et al., 2014, 2015; Onakpoya et al., 2019). Some clinical data suggest that opioids (e.g., tramadol, oxycodone, morphine) and neurointerventional surgery (e.g., nerve block, pulsed radiofrequency) are also effective (Binder and Baron, 2008; Argoff, 2011; Ke et al., 2013; Li et al., 2018), although the evidence is sparse, inconsistent, or lacking long-term follow-up (Petersen and Rowbotham, 2007; Whitley et al., 2010; Dworkin et al., 2013; Ke et al., 2013; McNicol et al., 2013). Nevertheless, oral drugs used to treat PHN can cause systemic and cognitive adverse effects (Nalamachu and Morley-Forster, 2012; Yu et al., 2016; Onakpoya et al., 2019). In addition, pain may last for years or a lifetime, and long-term medication is usually required. Patients with PHN are often older adults with underlying diseases who take other drugs, and patients who use oral drugs have an increased risk of adverse reactions and side effects (Schmader et al., 2010; Nalamachu and Morley-Forster, 2012). Therefore, identifying an effective treatment for PHN with fewer side effects is crucial.

Acupuncture therapy, an essential part of traditional Chinese medicine, is valuable in pain management (Vickers and Linde, 2014; Kelly and Willis, 2019) and has the advantages of quick effects and minimal adverse reactions (Melchart et al., 2004; Cui et al., 2022). One systematic review found insufficient evidence regarding whether acupuncture was more effective than pharmacological therapy but concluded that acupuncture was safer (Wang Y. et al., 2018). Nevertheless, another systematic review showed that the efficacy of acupuncture combined with cupping was significantly higher than that of conventional Western medicine therapies (Zhou et al., 2021). A systematic review by Wu et al. (2021) suggested that the efficacy of moxibustion was higher than that of control groups (pharmacological therapies, herbal medicine, or no treatment), despite the high heterogeneity of the included studies, and a systematic review by Pei et al. (2019) suggested that acupuncture is comparable to the effects of Western medicines, but with no reported adverse effects. Therefore, evidence-based medical evidence supports the efficacy of acupuncture-related therapies on PHN. Various treatments related to acupuncture may have different therapeutic effects, but no studies have compared them. The above factors contribute to the complexityin choosing clinical acupuncture treatment plans.

To compare the analgesic effects of various acupuncture therapies on PHN, we conducted a pairwise and network meta-analysis (NMA) to comprehensively and critically evaluate all available and eligible clinical evidence to provide evidence-based recommendations for selecting the best therapeutic acupuncture schedule. Additionally, we expound on the current insufficiency and prospects in the applications of acupuncture-related therapies, to provide a reference for subsequent research.

## 2. Methods

This study strictly followed the Preferred Reporting Items for Systematic Reviews and Meta-Analyses (PRISMA) (Moher et al., 2009) and PRISMA-NMA guidelines (Hutton et al., 2015). It was registered with the International Prospective Register of Systematic Reviews (CRD42021226422).

### 2.1. Data sources and search strategies

We searched eight electronic databases, including PubMed, Embase, Cochrane Library, Web of Science, Google Scholar, China National Knowledge Infrastructure, China Biomedical, Chongqing VIP, and Wan Fang, from inception to December 7, 2022; language was restricted to English and Chinese.

English search terms included: “neuralgia, postherpetic”, “postherpetic neuralgia”, “post-herpetic pain,”; “acupuncture analgesia”, “acupuncture therapy”, “acupuncture”, “manual acupuncture”, “surrounding acupuncture”, “surround needling”, “acupuncture, ear”, “auricular acupuncture”, “ear acupuncture”, “electroacupuncture”, “electro-acupuncture”, “fire needling”, “fire needle”, “warm needling”, “warm needle”, “moxibustion”, “thermal moxibustion”, “medicated thread moxibustion”, “pricking and cupping”, “pricking blood and cupping”, “blood-letting puncture and cupping”, “Fu’s subcutaneous needling”, “Fu’s acupuncture”, “acupoint catgut embedding”, “catgut embedding”, “dry needling”, “dry needle”, “laser acupuncture”.

Chinese search terms included: “带状疱疹后神经痛”, “带状疱疹后疼痛”, “针灸”, “针刺”, “围刺”, “耳针”, “耳穴”, “电针”, “火针”, “温针”, “艾灸”, “热敏灸”, “药线灸”, “刺络拔罐”, “刺血拔罐”, “梅花针”, “浮针”, “埋线”, “干针”, “激光针”.

The complete search strategy used for the Cochrane Central Register of Controlled Trials and other databases is shown in Table 1.

**TABLE 1:** CENTRAL: Search strategy.

Query	Search terms	Results	Date
*#1*	MeSH descriptor: [neuralgia, postherpetic] explode all trees	306	December 7, 2022
*#2*	(Postherpetic neuralgia): ti, ab, kw	1,145	December 7, 2022
*#3*	(Postherpetic pain):ti, ab, kw	279	December 7, 2022
*#4*	*#1 OR #2 OR #3*	1,189	December 7, 2022
*#5*	MeSH descriptor: [acupuncture analgesia] explode all trees	302	December 7, 2022
*#6*	MeSH descriptor: [acupuncture Therapy] explode all trees	5,393	December 7, 2022
*#7*	MeSH descriptor: [acupuncture] explode all trees	167	December 7, 2022
*#8*	MeSH descriptor: [acupuncture, ear] explode all trees	222	December 7, 2022
*#9*	MeSH descriptor: [electroacupuncture] explode all trees	900	December 7, 2022
*#10*	MeSH descriptor: [moxibustion] explode all trees	531	December 7, 2022
*#11*	(Acupuncture analgesia): ti, ab, kw	1,211	December 7, 2022
*#12*	(Acupuncture therapy): ti, ab, kw	9,588	December 7, 2022
*#13*	(Acupuncture): ti, ab, kw	17,576	December 7, 2022
*#14*	(Manual acupuncture): ti, ab, kw	665	December 7, 2022
*#15*	(Surrounding acupuncture): ti, ab, kw	63	December 7, 2022
*#16*	(Surrounding needling): ti, ab, kw	28	December 7, 2022
*#17*	(Auricular acupuncture): ti, ab, kw	861	December 7, 2022
*#18*	(Ear acupuncture): ti, ab, kw	751	December 7, 2022
*#19*	(Electroacupuncture): ti, ab, kw	3,118	December 7, 2022
*#20*	(Electro-acupuncture): ti, ab, kw	663	December 7, 2022
*#21*	(Fire needling): ti, ab, kw	57	December 7, 2022
*#22*	(Warm needling): ti, ab, kw	114	December 7, 2022
*#23*	(Fire needle): ti, ab, kw	133	December 7, 2022
*#24*	(Warm needle): ti, ab, kw	137	December 7, 2022
*#25*	(Moxibustion): ti, ab, kw	2,238	December 7, 2022
*#26*	(Thermal moxibustion): ti, ab, kw	43	December 7, 2022
*#27*	(Medicated thread moxibustion): ti, ab, kw	13	December 7, 2022
*#28*	(Pricking and cupping): ti, ab, kw	69	December 7, 2022
*#29*	(Pricking blood and cupping): ti, ab, kw	21	December 7, 2022
*#30*	(Blood-letting puncture and cupping): ti, ab, kw	26	December 7, 2022
*#31*	(Fu’s subcutaneous needling): ti, ab, kw	35	December 7, 2022
*#32*	(Fu’s acupuncture): ti, ab, kw	52	December 7, 2022
*#33*	(Acupoint catgut embedding): ti, ab, kw	158	December 7, 2022
*#34*	(Catgut embedding): ti, ab, kw	203	December 7, 2022
*#35*	(Dry needling): ti, ab, kw	931	December 7, 2022
*#36*	(Dry needle): ti, ab, kw	421	December 7, 2022
*#37*	(Laser acupuncture): ti, ab, kw	675	December 7, 2022
*#38*	#5 OR #6 OR #7 OR #8 OR #9 OR #10 OR #11 OR #12 OR #13 OR #14 OR #15 OR #16 OR #17 OR #18 OR #19 OR #20 OR #21 OR #22 OR #23 OR #24 OR #25 OR #26 OR #27 OR #28 OR #29 OR #30 OR #31 OR #32 OR #33 OR #34 OR #35 OR #36 OR #37	20,699	December 7, 2022
*#39*	*#4 AND #38 (in trials)*	81	December 7, 2022

ti, title; ab, abstract; kw, keywords.

Additionally, we searched the World Health Organization International Clinical Trial Registration platform,^1^ China Clinical Trial Registration Center,^2^ previous meta-analyses, and gray literature.^3^

Two researchers crosschecked the search results and searched the reference lists of the included studies.

### 2.2. Eligibility criteria

We predefined inclusion criteria following the PICOS protocol (Cumpston et al., 2019):

(1) Participants: to address the inconsistent diagnostic criteria for PHN are inconsistent, we referred to the International Association for the Study of Pain Classification of Chronic Pain for the International Classification of Diseases-11, which defines PHN as pain persisting for over 3 months after the onset of rash (Scholz et al., 2019), and the Chinese Expert Consensus on Diagnosis and Treatment of Postherpetic Neuralgia, which defines PHN as pain persisting for over 1 month after the rash healed (Yu et al., 2016). Patients who met either of the above criteria were included, regardless of age, sex, or race.
(2) Treatment group: treatment groups involving a single type of acupuncture combined with pharmacotherapy were included. Acupuncture techniques were defined as manual, electro- (EA), and other acupuncture used in clinical practice (e.g., fire needling and warm needling); however, acupoint injections were excluded. No limitations on needle material, acupoint matching, duration of needle retention, and treatment course were imposed.
(3) Control group: controls received pharmacotherapy, a different single type of acupuncture than the treatment group, or sham acupuncture. To ensure homogeneity, pharmacotherapies were limited to evidence-based effective agents for PHN, including antiepileptics (pregabalin, gabapentin), tricyclic antidepressants (e.g., amitriptyline, nortriptyline), opioids (morphine, oxycodone, and tramadol), and topical treatments (5% lidocaine or capsaicin patches) (Johnson and Rice, 2014; Song et al., 2018). When antiepileptics were used, mecobalamin could be used as adjunctive therapy, reducing the adverse events of antiepileptics with no significant effect on pain relief. The same drug should be administered to the treatment and control groups when a combination of pharmacotherapy is used.
(4) Outcome indicators: primary outcome was reduced pain intensity, indicated by the change from baseline. The pain was measured using a visual analog scale (VAS) or numerical rating scale. Secondary outcomes were improvement in pain-related outcomes in Pittsburgh Sleep Quality Index (PSQI) score, Self-Rating Depression Scale (SDS) score, clinical response rate (defined as the ratio of the number of effective cases to the total number of patients), quality of life, and adverse events (AEs).
(5) Study design: studies based on needle insertion or stimulation of acupoints for therapeutic purposes, including randomized controlled trials (RCTs) that evaluated acupuncture and related therapies for PHN, were included.

Exclusion criteria were:

(1) Secondary analysis or duplicate publication (with multilingual publications, only the earliest was chosen).
(2) Full text was unavailable after contacting the corresponding author.
(3) Flawed study design, such as not strictly following the principle of randomization (e.g., randomization performed based on the order in which patients were admitted to the hospital, odd and even case numbers, or birth dates).
(4) Missing baseline data.
(5) Unspecified diagnostic criteria.
(6) Outcome indicators are unobtainable or not applicable to the NMA.
(7) Interventions included acupuncture combined with other acupuncture therapies.
(8) Interventions were single drug treatment with no evidence-based efficacy (e.g., antiviral drugs, Chinese herbal medicine) (Johnson and Rice, 2014; Song et al., 2018).

### 2.3. Trial selection

After deleting duplicate records using EndNote X9 (Clarivate Analytics, London, UK), two researchers independently reviewed all titles and abstracts based on the inclusion and exclusion criteria; studies that did not meet the inclusion criteria were excluded. Subsequently, full texts of the studies that met the inclusion criteria were carefully reviewed to determine if they should be included. Differences of opinion were resolved through discussions. If no agreement could be reached, a decision arbitrator was consulted. All researchers are licensed physicians with more than 2 years of clinical acupuncture experience.

### 2.4. Data extraction

The following information was extracted according to a fixed protocol. After the final selection, two researchers extracted the data independently and created summary tables. Data were extracted according to a template, including the study (first author, publication year, location) and participant characteristics (patient source, sample size, mean age, PHN course), intervention (type, acupoint, specific drug, dose, frequency, treatment course), and outcome indicators. The results were then cross-checked.

### 2.5. Quality assessment

Under the Cochrane Collaboration recommendation (Cumpston et al., 2019), two researchers independently assessed these seven major risks of bias for all included studies using RevMan version 5.4 (The Cochrane Collaboration, London, UK): random sequence generation, allocation hiding, blinding of participating researchers, blinding of outcome evaluation, incomplete outcome data, selective reporting, and other types of bias. Outcomes were classified into (as high, low, or unclear risk).

### 2.6. Data synthesis and analysis

A pairwise meta-analysis was performed using STATA software version 15.1 (StataCorp LLC, College Station, TX, US). Weighted mean differences (WMDs) and corresponding 95% confidence intervals (CIs) were used to analyze continuous variables. *P* < 0.05 indicated statistical significance. A fixed-effects model was used for analysis (obvious heterogeneity; I^2^ < 50%); otherwise, a random-effects model was used.

The similarity hypothesis is one of the most important assumptions in NMA, which states that factors affecting the effect size are similar among all studies and different control groups. Only when the similarity hypothesis is satisfied can NMA produce reliable results; otherwise, bias is present. In this study, descriptive statistical analysis was performed on the RCTs’ population characteristics, and participants’ baseline levels were evaluated, including mean age and disease duration. Line graphs were drawn for visualization.

A Bayesian multiple treatment NMA was performed using R (version 4.0.5; R Foundation for Statistical Computing, Vienna, Austria) and the “gemtc” package, which interfaces with OpenBUGS. Network plots were created in STATA (StataCorp LLC). A random-effects model was computed using Markov chain Monte Carlo (Gelman and Rubin, 1996) methods, based on simulations of 50,000 iterations in each of the four chains. The node-splitting method was used to test the consistency of the assumptions (Dias et al., 2010). *P* > 0.05, indicated that direct and indirect comparisons had better consistency. A convergence diagnosis plot was used to monitor the convergence of the model (Gelman and Rubin, 1996) by calculating the median value of the shrink factor and the value of 97.5% in each iteration and observing the consistency of the curves after 50,000 iterations. The surface under the cumulative ranking (SUCRA) curve was used for probability sorting (Salanti et al., 2011). A rank histogram was drawn, and the SUCRA value was 0 when the intervention was 0 or 1 for the worst and best intervention, respectively. Finally, publication bias was tested using comparison-adjusted funnel plots in STATA (StataCorp LLC).

## 3. Results

### 3.1. Literature retrieval and characteristics

**FIGURE 1:**
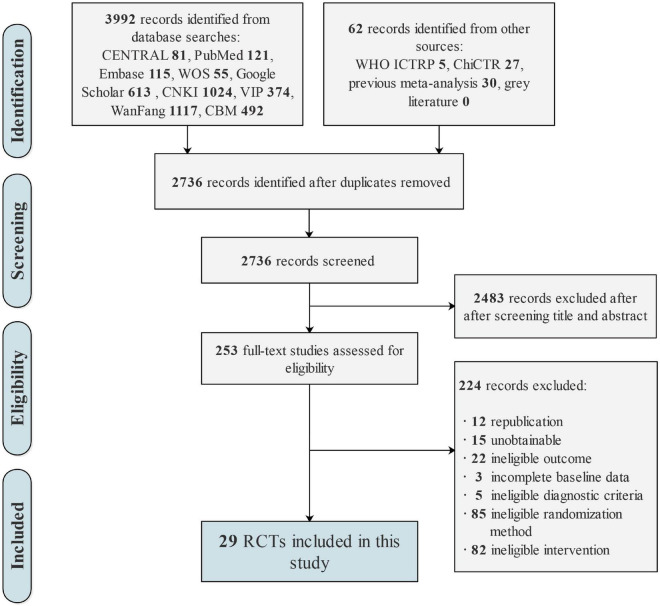
Flow chart of study selection.

The initial search resulted in 3,992 pending records, and we selected 253 potentially eligible articles for full-text review. After further screening, 224 studies were excluded; the reasons for excluding each study are detailed in Supplementary Appendix 1. Ultimately, 29 RCTs (Wang, 2006; Huang et al., 2011; Chen et al., 2012, 2018; Tian, 2013; Xie and Wen, 2013; Zhang, 2013, 2015; Duan, 2014; Sheng et al., 2014; Lei, 2015; Sun and Li, 2015; Hong, 2016; Wu and Zhao, 2016; Yang and Pan, 2016; Han et al., 2018; Xie et al., 2018; Xu, 2018, 2020; Zhou and Huang, 2018; Chen, 2019; Mo, 2019; Zhong, 2019; Pang, 2020; Wang et al., 2020; Zhang H. L. et al., 2020; Zhao et al., 2020; Ding et al., 2021; Zhang et al., 2021) met the inclusion criteria and were included in the quality evaluation and statistical analysis; all Chinese references for the included trials are detailed in Supplementary Appendix 2. The flowchart illustrating the selection process is shown in Figure 1.

All included studies were conducted in China, published in Chinese, and included a total of 1,973 patients (mean age: 46.91–66.79 years); 54.45% (*n* = 1010) were women. The average sample size of included studies was 64 (range: 37–115), and included seven types of acupuncture-related therapies: manual acupuncture, EA, fire needling, pricking and cupping, Fu’s acupuncture, medicated thread moxibustion, and acupoint catgut embedding in addition to pharmacotherapy. Only comparisons between acupuncture-related therapies and antiepileptic drugs were found for the pharmacotherapy group, and no eligible studies compared acupuncture with tricyclic antidepressants, opioids, or topical treatments. The Ashi point (82.76%) was the most frequently used acupoint among the included studies, followed by the Jiaji point (51.72%). Interestingly, pain reduction in all studies was reported according to a VAS. The details of each trial are provided in Table 2.

**TABLE 2:** Characteristics of the included studies.

References	Sample size (female rate)	Group	Types of intervention	Details of intervention (specific drug/acupoint, frequency, course of treatment)	Follow-up (weeks)	Age (years)	Duration of PHN (months)	Outcome indicator	Cochrane Collaboration Risk of Bias
Wang, 2006	34 (58.8%)	Treatment	Fire needling	Ashi point, Jiaji point, qod; 14 days	1&4	60.06 ± 7.34	3.63 ± 2.31	① ④	Low; unclear; unclear; unclear; low; low; unclear
	34 (52.9%)	Control	Manual acupuncture	Ashi point, Jiaji point, qod; 14 days		59.91 ± 7.46	3.75 ± 2.77		
Huang et al., 2011	49 (42.9%)	Treatment	Pricking and cupping	Ashi point, GB21, qod; 30 days	/	64.28	2.93	① ④	Low; unclear; unclear; unclear; unclear; unclear; unclear
	47 (46.8%)	Control	Manual acupuncture	Ashi point, LI4, LI11, LR3, SP6, qod; 30 days		65.35	3.45		
Chen et al., 2012	35 (51.4%)	Treatment	Medicated thread moxibustion	Ashi point, LI10, PC6, SP6, ST36, LR3, qd; 14 days	/	65.81 ± 4.45	6.98 ± 2.58	① ④	Low; unclear; unclear; unclear; unclear; unclear; unclear
	35 (54.3%)	Control	Fire needling	Ashi point, qd; 14 days		66.31 ± 4.05	7.12 ± 2.54		
Zhang, 2013	26 (50.0%)	Treatment	Electroacupuncture	Ashi point, LI4, LJ6, LR3, ST36, qd; 20 days	4	56.30 ± 12.54	4.5	① ③ ④ ⑤	Low; low; high; low; low; low; unclear
	27 (55.6%)	Control	Antiepileptics	Gabapentin, maximum dose 3600 mg per day; 20 days		57.55 ± 7.69	4.4		
Tian, 2013	32 (56.3%)	Treatment	Pricking and cupping	Ashi point, qod; 16 days	/	61.15 ± 7.13	3.56 ± 2.68	① ③ ④ ⑤	Low; high; unclear; unclear; low; low; unclear
	32 (50.0%)	Control	Antiepileptics	Pregabalin, 150mg, bidpo; 16 days		60.93 ± 7.45	3.61 ± 2.12		
Xie and Wen, 2013	35 (45.7%)	Treatment	Manual acupuncture	Ashi point, Jiaji point, SP6, ST36, KI3, SP9, 20 min, qd; 21 days	4	60.5 ± 8.2	5.10 ± 3.30	① ② ④	Low; unclear; unclear; unclear; low; low; unclear
	30 (34.3%)	Control	Electroacupuncture	Ashi point, Jiaji point, SP6, ST36, KI3, SP9, 3/90 Hz dilatational wave, 20 min, qd; 21 days		61.2 ± 7.8	4.90 ± 3.50		
Duan, 2014	30 (56.7%)	Treatment	Electroacupuncture	Ashi point, Jiaji point, 30 mins, bid; 18 days	4	61.30 ± 8.35	5.83 ± 10.0	① ④	Low; unclear; unclear; unclear; low; low; unclear
	30 (60.0%)	Control	Manual acupuncture	Ashi point, Jiaji point, 30 min, bid; 18 days		62.20 ± 9.76	5.90 ± 9.29		
Sheng et al., 2014	20 (55.5%)	Treatment	Electroacupuncture	Ashi point, Jiaji point, 30 min, bid; 18 days	/	46 to 77	3 to 8	① ④	Low; unclear; unclear; unclear; unclear; unclear; unclear
	20 (65.5%)	Control	Manual acupuncture	Ashi point, Jiaji point, 30 min, bid; 18 days		42 to 80	3 to 6		
Sun and Li, 2015	45 (57.8%)	Treatment	Electroacupuncture	Ashi point, Jiaji point, 30 mins, qd; 10 days	/	20 to 69	1 to 7	① ④	Low; unclear; unclear; unclear; low; low; unclear
	45 (51.1%)	Control	Manual acupuncture	Ashi point, Jiaji point, 30 mins, qd; 10 days		33 to 58	2 to 9		
Zhang, 2015	20 (60.0%)	Treatment	Fire needling	Ashi point, qod; 30 days	4	60.0 ± 7.0	3.62 ± 0.54	① ④ ⑤	Low; high; unclear; unclear; low; low; unclear
	20 (35.5%)	Control	Manual acupuncture	Ashi point, 30 min, qd; 30 days		60.0 ± 7.0	3.76 ± 0.43		
	20 (40.0%)	Control	Pricking and cupping	Ashi point, qod; 30 days		61.0 ± 7.0	3.59 ± 0.68		
	20 (55.0%)	Control	Antiepileptics	Pregabalin, 150mg, bidpo; 30 days		60.0 ± 8.0	3.65 ± 0.51		
Lei, 2015	20 (75.0%)	Treatment	Electroacupuncture plus Antiepileptics	Ashi point, SJ6, GB34, ST36 30 min, 5 times per 7 days plus Gabapentin; 14 days	4	62.55 ± 7.48	3.1	① ② ③ ④ ⑤	Low; unclear; unclear; unclear; low; low; unclear
	20 (55.0%)	Control	Electroacupuncture	Ashi point, SJ6, GB34, 30 min, 5 times per 7 days; 14 days		64.20 ± 10.81	3.2		
	19 (42.1%)	Control	Antiepileptics	Gabapentin, maximum dose 1800 mg per day; 14 days		66.79 ± 11.25	3		
Yang and Pan, 2016	34 (35.3%)	Treatment	Electroacupuncture plus Antiepileptics	Ashi point, Jiaji point, 50 Hz, 30 min, qd plus Gabapentin; 28 days	4	56.8 ± 14.9	4.90 ± 3.80	① ④ ⑤	Low; unclear; unclear; unclear; low; low; unclear
	34 (32.4%)	Control	Antiepileptics	Gabapentin, maximum dose 1800 mg per day; 28 days		54.3 ± 12.4	5.60 ± 3.40		
Wu and Zhao, 2016	25 (64.0%)	Treatment	Fu’s subcutaneous needling	Ashi point; 14 days	/	40 to 70	2 to 8	① ⑤	Low; high; unclear; unclear; unclear; unclear; unclear
	25 (56.0%)	Control	Antiepileptics	Pregabalin, 150 mg, bidpo; 14 days		38 to 70	2 to 7		
Hong, 2016	29	Treatment	Pricking and cupping	Ashi point, qod; 28 days	/	61	5.1	① ⑤	Low; unclear; unclear; unclear; low; low; unclear
	29	Control	Antiepileptics	Gabapentin, maximum dose 1800 mg per day; 28 days		61	5.3		
Zhou and Huang, 2018	30	Treatment	Fire needling plus Antiepileptics	BL15, BL17, qod plus Pregabalin; 10 days	/	52.78 ± 8.12	6.85 ± 4.48	① ④	Low; unclear; unclear; unclear; unclear; unclear; unclear
	30	Control	Antiepileptics	Pregabalin, 150 mg, bidpo; 10 days		53.34 ± 7.60	6.62 ± 4.13		
Xu, 2018	34 (58.8%)	Treatment	Fire needling	Ashi point, qod; 14 days	1&4	60.06 ± 7.34	3.63 ± 2.31	① ④ ⑤	Low; unclear; unclear; unclear; low; low; unclear
	34 (52.9%)	Control	Manual acupuncture	Ashi point, Jiaji point, 30 min, qd; 14 days		59.91 ± 7.46	3.75 ± 2.77		
Xie et al., 2018	40 (47.5%)	Treatment	Electroacupuncture plus antiepileptics	Ashi point, Jiaji point, 50 Hz, 60 min, 1 time per 3 days plus Gabapentin; 42 days	/	65.3 ± 10.4	5.15	① ④ ⑤	Low; unclear; unclear; unclear; unclear; unclear; unclear
	40 (55.0%)	Control	Antiepileptics	Gabapentin, maximum dose 1800 mg per day; 42 days		65.8 ± 10.8	5.50		
Chen et al., 2018	45 (31.1%)	Treatment	Fire needling	Ashi point, Jiaji point, qod; 20 days	/	61.24 ± 3.65	3.42 ± 0.39	① ② ④	Low; unclear; unclear; unclear; unclear; unclear; unclear
	45 (32.0%)	Control	Manual acupuncture	Ashi point, Jiaji point, 30 min, qd; 20 days		61.15 ± 3.74	3.35 ± 0.37		
Han et al., 2018	32 (50.0%)	Treatment	Pricking and cupping	Jiaji point, 15 min, 1 time per 3 days; 60 days	/	46.91 ± 13.88	3.90 ± 3.70	①	Low; low; unclear; low; unclear; unclear; low
	32 (53.1%)	Control	Antiepileptics	Gabapentin, maximum dose 1800 mg per day; 60 days		47.91 ± 13.94	3.2 ± 4.10		
Mo, 2019	19 (52.6%)	Treatment	Pricking and cupping	Jiaji point, 10 min, 1 time per 3 days; 60 days	/	47.05 ± 12.75	2.9	① ④	Low; low; unclear; unclear; low; low; unclear
	18 (55.6%)	Control	Antiepileptics	Gabapentin, maximum dose 1800 mg per day; 60 days		48.17 ± 11.17	2.8		
Zhong, 2019	33 (51.5%)	Treatment	Fire needling	Ashi point, Jiaji point, qod; 28 days	/	60.03 ± 3.09	3.61 ± 1.00	① ② ③ ④ ⑤	Low; low; unclear; unclear; low; low; unclear
	33 (54.5%)	Control	Antiepileptics	Pregabalin, 75 mg, bidpo; 28 days		59.55 ± 5.36	3.58 ± 1.00		
Chen, 2019	30 (50.0%)	Treatment	Catgut embedding plus antiepileptics	Jiaji point, 1 time per 14 days plus Antiepileptics; 28 days	/	55.17 ± 10.88	6.23 ± 1.61	① ② ④	Low; unclear; unclear; unclear; low; low; unclear
	28 (53.6%)	Control	Antiepileptics	Pregabalin, 75 mg, bidpo; 28 days		52.93 ± 10.04	6.41 ± 1.92		
Pang, 2020	34 (55.9%)	Treatment	Fire needling plus antiepileptics	Wrist ankle point, 1 time per 4 days plus Gabapentin; 20 days	4	60	3	① ② ④ ⑤	Low; low; high; low; low; low; low
	33 (45.5%)	Control	Antiepileptics	Gabapentin, maximum dose 900 mg; 20 days		60	3		
Zhang et al., 2020	54 (46.3%)	Treatment	Fire needling	Ashi point, qod; 14 days	/	54.1 ± 13.7	6.20 ± 2.60	① ④	Low; high; unclear; unclear; low; low; unclear
	53 (49.1%)	Control	Fu’s subcutaneous needling	Ashi point, qod; 14 days		58.3 ± 11.4	6.30 ± 2.70		
Xu, 2020	29 (55.2%)	Treatment	Manual acupuncture	Regulating mind points, 30 min, qd; 14 days	4&12	50.55 ± 10.02	5.06 ± 2.39	① ④	Low; high; unclear; unclear; low; low; unclear
	28 (50.0%)	Control	Electroacupuncture	Ashi point, Jiaji point, dilatational wave, 2/100 Hz, 30 min, qd; 14 days		53.07 ± 9.49	5.54 ± 2.83		
Wang et al., 2020	34 (51.4%)	Treatment	Pricking and cupping	Ashi point, 20 min, qod; 16 days	/	40 to 70	3.09	① ④	Low; unclear; unclear; unclear; low; low; unclear
	32 (54.3%)	Control	Antiepileptics	Gabapentin, maximum dose 900 mg; 16 days		40 to 69	3.06		
Zhao et al., 2020	28 (60.7%)	Treatment	Pricking and cupping	Ashi poinrt, 30 min, qod; 40 days	/	65.45 ± 2.23	6.02 ± 2.50	① ② ④ ⑤	Low; unclear; unclear; unclear; unclear; unclear; unclear
	28 (64.3%)	Control	Manual acupuncture	Ashi poinrt, 5 min, qod; 40 days		65.67 ± 2.32	5.79 ± 2.45		
Ding et al., 2021	57 (56.1%)	Treatment	Pricking and cupping plus antiepileptics	Ashi point, Jiaji point, 2 to 3 times per 7 days plus Gabapentin plus Mecobalamine 0.5 mg tidpo; 28 days	/	59.33 ± 6.25	3.71 ± 0.49	① ④ ⑤	Low; unclear; unclear; unclear; unclear; unclear; unclear
	58 (53.4%)	Control	Antiepileptics	Gabapentin, maximum dose 1800 mg per day plus Mecobalamine 0.5 mg, tidpo; 28 days		62.45 ± 9.23	3.49 ± 0.63		
Zhang et al., 2021	31 (48.4%)	Treatment	Pricking and cupping plus antiepileptics	Ashi point, 20 min, qod plus Pregabalin; 30 days	/	61.69 ± 8.43	7.43 ± 1.49	① ④ ⑤	Low; unclear; unclear; unclear; unclear; unclear; unclear
	30 (43.3%)	Control	Antiepileptics	Pregabalin, 150 mg, bidpo; 28 days		61.42 ± 7.96	7.68 ± 1.52		

① = VAS; ② = PSQI; ③ = SDS; ④ = response rate; ⑤ = adverse events.

### 3.2. Study quality assessment

Twenty-nine studies implemented the correct randomization method. Five studies (Tian, 2013; Zhang, 2015; Wu and Zhao, 2016; Xu, 2020; Zhang H. L. et al., 2020) were classified as high-risk for allocation concealment because they sequentially assigned patients to treatment in the order of inpatient admissions after patients were correctly randomly assigned to treatment or control groups, which increased the possibility of exposure allocation information. Three studies (Zhang, 2013; Han et al., 2018; Pang, 2020) performed outcome measure blindness, whereas the remaining studies did not. Eighteen studies (Wang, 2006; Tian, 2013; Xie and Wen, 2013; Zhang, 2013, 2015; Duan, 2014; Lei, 2015; Sun and Li, 2015; Hong, 2016; Yang and Pan, 2016; Xu, 2018, 2020; Chen, 2019; Mo, 2019; Zhong, 2019; Pang, 2020; Wang et al., 2020; Zhang H. L. et al., 2020) reported detailed information on shedding and loss to follow-up. Regarding other biases, no studies were classified as high-risk. Table 2 summarizes all included studies’ quality assessments and Cochrane risk of bias (Supplementary Appendix 3).

### 3.3. Pairwise meta-analysis

Pairwise meta-analyses were performed to compare the two interventions with a combined effect size. The results are shown in Tables 3–5. Compared with antiepileptics for PHN, manual acupuncture (one RCT; WMD = 1.73, 95% CI = 0.10–2.47, *P* = 0.000 < 0.001), EA (two RCTs; WMD = 1.50, 95% CI = 1.03–1.97, *P* = 0.000 < 0.001), fire needling (two RCTs; WMD = 1.42, 95% CI = 0.91–1.93, *P* = 0.000 < 0.001), pricking and cupping (six RCTs; WMD = 1.50, 95% CI = 0.52–2.47, *P* = 0.003 < 0.01), Fu’s acupuncture (one RCT; WMD = 0.83, 95% CI = 0.80–2.86, *P* = 0.000 < 0.001), EA plus antiepileptics (three RCTs; WMD = 1.79, 95% CI = 1.03–2.56, *P* = 0.000 < 0.001), fire needling plus antiepileptics (two RCTs; WMD = 1.47, 95% CI = 1.11–1.84, *P* = 0.000 < 0.001), pricking and cupping plus antiepileptics (two RCTs; WMD = 2.12, 95% CI = 1.77–2.48, *P* = 0.000 < 0.001), and acupoint catgut embedding plus antiepileptics (one RCT; WMD = 0.66, 95% CI = 0.18–1.14, *P* = 0.007 < 0.01) were all more efficacious in reducing VAS scores. However, EA plus antiepileptics had a greater effect than EA alone (one RCT; WMD = 1.25, 95% CI = 0.24–2.26, *P* = 0.015 < 0.05). Nevertheless, in terms of VAS score reduction, no significant differences were observed between manual acupuncture and fire needling, manual acupuncture and pricking and cupping, medicated thread moxibustion and fire needling, and pricking and cupping and fire needling. No significant differences were observed between EA and antiepileptics in reducing PSQI (Table 4) or SDS scores (Table 5), or between manual acupuncture and pricking and cupping in reducing PSQI scores (Table 4). Tables 3–5 present the results of the pairwise meta-analysis and heterogeneity.

**TABLE 3:** Visual analog scale (VAS) pairwise meta-analysis results.

Comparison	Number	WMD [95% CI]	I^2^ (%)	*P*
B VS. A	1	**1.73 [0.10, 2.47]**	–	0
C VS. A	2	**1.50 [1.03, 1.97]**	0	0
D VS. A	2	**1.42 [0.91, 1.93]**	0	0
*E VS. A	6	**1.50 [0.52, 2.47]**	95.1	0.003
F VS. A	1	**1.83 [0.80, 2.86]**	–	0
*H VS. A	3	**1.79 [1.03, 2.56]**	75.5	0
I VS. A	2	**1.47 [1.11, 1.84]**	0	0
J VS. A	2	**2.12 [1.77, 2.48]**	0	0
K VS. A	1	**0.66 [0.18, 1.14]**	–	0.007
*D VS. B	4	0.25 [−0.25, 0.75]	52.6	0.323
*E VS. B	3	0.83 [−0.19, 1.85]	85.0	0.112
H VS. C	1	**1.25 [0.24, 2.26]**	–	0.015
F VS. D	1	**0.42 [0.08, 0.77]**	–	0.017
G VS. D	1	0.37 [−0.68, 1.42]	–	0.488
D VS. E	1	0.05 [−0.70, 0.80]	–	0.897

The bold font indicates a statistical difference; *random effect model was used; A = antiepileptics; B = manual acupuncture; C = electroacupuncture; D = fire needling; E = pricking and cupping; F = Fu’s acupuncture; G = medicated thread moxibustion; H = electroacupuncture plus antiepileptics; I = fire needling plus antiepileptics; J = pricking and cupping plus antiepileptics; K = acupoint catgut embedding plus antiepileptics.

**TABLE 4:** Pittsburgh Sleep Quality Index (PSQI) pairwise meta-analysis results.

Comparison	Number	WMD [95% CI]	I^2^ (%)	*P*
C VS. A	1	1.04 [−0.51, 2.59]	–	0.190
D VS. A	1	**2.28 [1.51, 3.06]**	–	0
H VS. A	1	**4.64 [3.16, 6.12]**	–	0
I VS. A	1	**2.76 [1.77, 3.75]**	–	0
K VS. A	1	**1.60 [0.29, 2.91]**	–	0.017
C VS. B	1	−**1.79 [**−**2.96,**−**0.62]**	–	0.003
D VS. B	1	**0.40 [0.06, 0.74]**	–	0.021
E VS. B	1	1.63 [−0.05, 3.31]	–	0.058
H VS. C	1	**3.60 [2.06, 5.14]**	–	0

The bold font indicates a statistical difference; A = antiepileptics; B = manual acupuncture; C = electroacupuncture; D = fire needling; E = pricking and cupping; H = electroacupuncture plus antiepileptics; I = fire needling plus antiepileptics; K = acupoint catgut embedding plus antiepileptics.

**TABLE 5:** Self-Rating Depression Scale (SDS) pairwise meta-analysis results.

Comparison	Number	WMD [95% CI]	I^2^ (%)	*P*
*C VS. A	2	6.73 [−3.61, 17.07]	88.1	0.202
D VS. A	1	**10.37 [8.95, 11.79]**	–	0
E VS. A	1	**12.24 [8.64, 15.84]**	–	0
H VS. A	1	**10.09 [4.19, 15.99]**	–	0.001
H VS. C	1	**9.15 [1.33, 16.97]**	–	0.022

The bold font indicates a statistical difference; *random effect model was used; A = antiepileptics; C = electroacupuncture; D = fire needling; E = pricking and cupping; H = electroacupuncture plus antiepileptics.

### 3.4. Network meta-analysis

**FIGURE 2:**
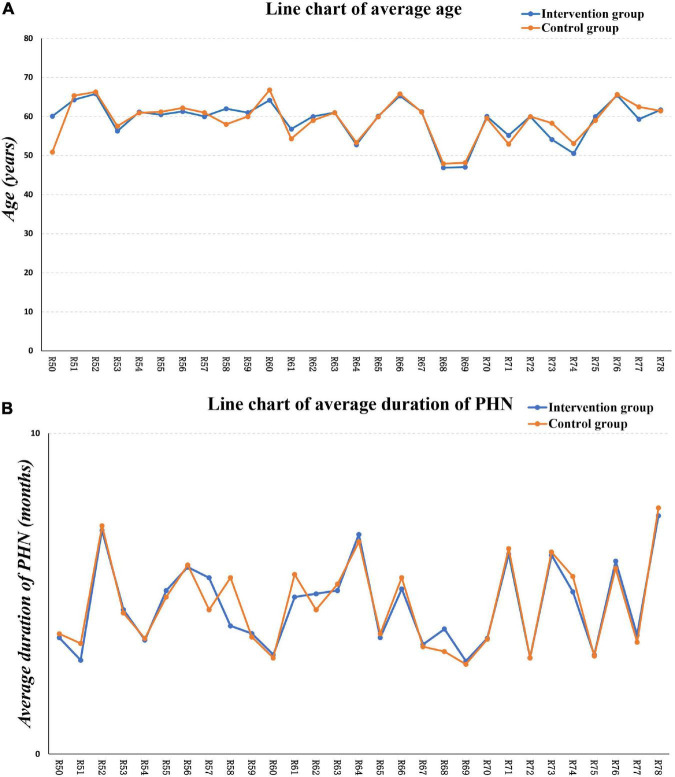
Baseline assessment. **(A)** The average age assessment, **(B)** the average PHN duration assessment.

The similarity was evaluated by assessing the baseline differences in the enrolled patients’ mean age and disease duration. As shown in Figure 2A, the average patient age in various studies showed few differences and high degrees of similarity. As shown in Figure 2B, the disease course of patients in various studies showed few differences and high degrees of similarity, satisfying the similarity assumption and obtaining reliable NMA results.

**FIGURE 3:**
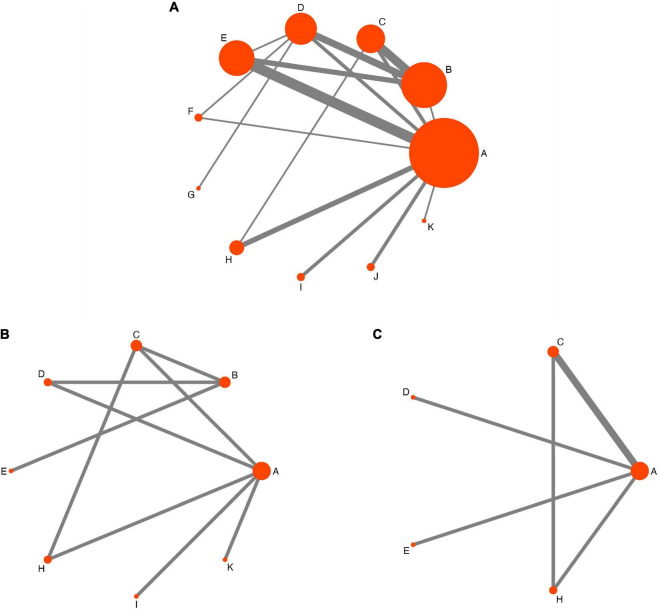
Network evidence diagram. A = antiepileptics; B = manual acupuncture; C = electroacupuncture; D = fire needling; E = pricking and cupping; F = Fu’s acupuncture; G = medicated thread moxibustion; H = electroacupuncture plus antiepileptics; I = fire needling plus antiepileptics; J = pricking and cupping plus antiepileptics; K = acupoint catgut embedding plus antiepileptics. **(A)** The visual analog scale, **(B)** the Pittsburgh Sleep Quality Index (PSQI), and **(C)** the Self-Rating Depression Scale (SDS) network evidence diagram.

Figure 3 shows the NMA network evidence diagrams. All included studies with 1,973 participants and 11 therapy techniques had VAS data (Figure 3A), including one three-arm (Lei, 2015) and one four-arm (Zhang, 2015) study; the remaining studies were two-arm RCTs. Of these, the largest sample size was in the antiepileptic group, and studies comparing pricking and cupping with antiepileptics were the most common. PSQI scores were reported in seven studies (Xie and Wen, 2013; Lei, 2015; Chen et al., 2018; Chen, 2019; Zhong, 2019; Pang, 2020; Zhao et al., 2020) involving 461 participants and eight interventions (Figure 3B). Four studies (Tian, 2013; Zhang, 2013; Lei, 2015; Zhong, 2019) with 242 participants and five therapies reported SDS scores. Similarly, most participants were in the antiepileptic group (Figure 3C).

Effective NMA results depend on the internal consistency of the evidence network; direct evidence and various sources of indirect evidence should be consistent (Chaimani et al., 2013). We used the node-splitting method was used to test inconsistencies in the NMA. The results (Supplementary Appendix 4) showed that direct or indirect comparisons of each segmentation node had no statistical significance (*P* > 0.05), which demonstrated no evidence of inconsistency. A convergence diagnosis plot was used to test the convergence of the model. The results showed that the shrink factor’s median and 97.5% value tended to be 1 and stabilized after 50,000 iterations, with continued stability of the curve fitting and merging, indicating that the convergence of the model was excellent (Supplementary Appendix 5).

**FIGURE 4:**
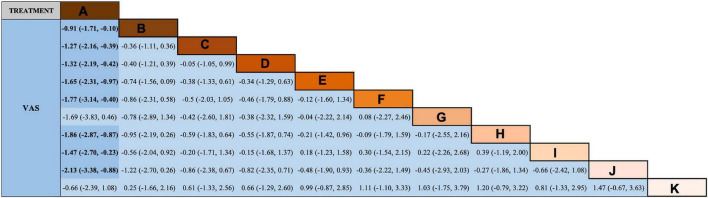
Network meta-analysis (NMA) results of visual analog scale (VAS). A = antiepileptics; B = manual acupuncture; C = electroacupuncture; D = fire needling; E = pricking and cupping; F = Fu’s acupuncture; G = medicated thread moxibustion; H = electroacupuncture plus antiepileptics; I = fire needling plus antiepileptics; J = pricking and cupping plus antiepileptics; K = acupoint catgut embedding plus antiepileptics.

**FIGURE 5:**
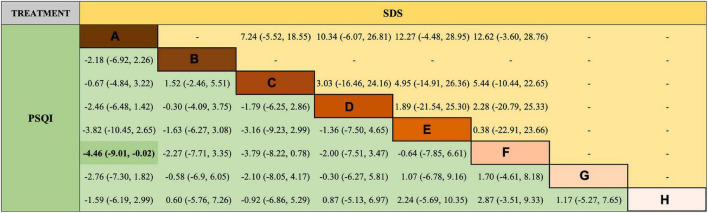
Network meta-analysis results of Pittsburgh Sleep Quality Index (PSQI) and Self-Rating Depression Scale (SDS). A = antiepileptics; B = manual acupuncture; C = electroacupuncture; D = fire needling; E = pricking and cupping; H = electroacupuncture plus antiepileptics; I = fire needling plus antiepileptics; K = acupoint catgut embedding plus antiepileptics.

Regarding VAS score reduction, the results (Figure 4) show that all therapy techniques were advantageous over antiepileptics alone, except medicated thread moxibustion, acupoint catgut embedding plus antiepileptics. Additionally, EA plus antiepileptics was superior to antiepileptics alone in reducing PSQI scores (Figure 5).

**FIGURE 6:**
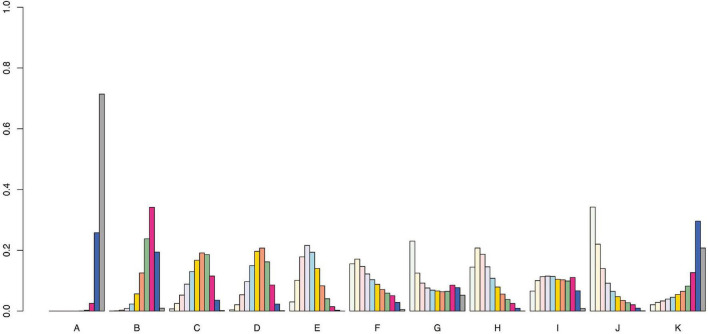
Visual analog scale probability ranking diagram. A = antiepileptics; B = manual acupuncture; C = electroacupuncture; D = fire needling; E = pricking and cupping; F = Fu’s acupuncture; G = medicated thread moxibustion; H = electroacupuncture plus antiepileptics; I = fire needling plus antiepileptics; J = pricking and cupping plus antiepileptics; K = acupoint catgut embedding plus antiepileptics.

**FIGURE 7:**
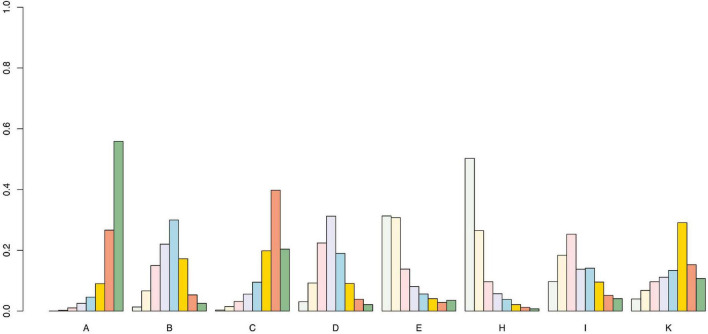
Pittsburgh Sleep Quality Index probability ranking diagram. A = antiepileptics; B = manual acupuncture; C = electroacupuncture; D = fire needling; E = pricking and cupping; H = electroacupuncture plus antiepileptics; I = fire needling plus antiepileptics; K = acupoint catgut embedding plus antiepileptics.

**FIGURE 8:**
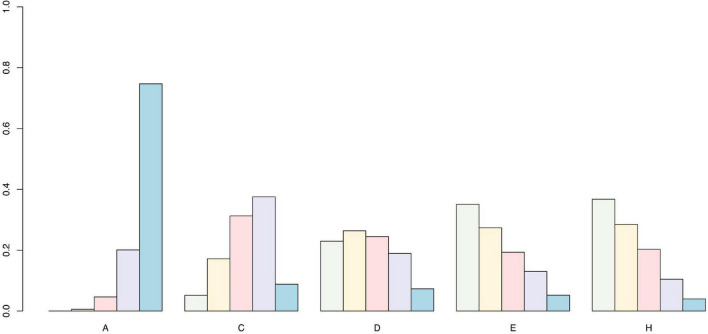
Self-Rating Depression Scale probability ranking diagram. A = antiepileptics; C = electroacupuncture; D = fire needling; E = pricking and cupping; H = electroacupuncture plus antiepileptics.

We used the SUCRA curve for probability sorting to plot probability sorting histograms (Figures 6–8) using R software. Figure 6 shows that antiepileptic drugs ranked lowest for VAS score reduction. Of the 11 treatments, the top three techniques for VAS score reduction were: pricking and cupping plus antiepileptics, EA plus antiepileptics, and pricking and cupping. While EA plus antiepileptics, pricking and cupping, and fire needling plus antiepileptics were the three best interventions for reducing PSQI scores (Figure 7). Among the five therapies, EA plus antiepileptics, pricking and cupping, and EA ranked highest in reducing SDS scores (Figure 8).

### 3.5. Adverse events

**FIGURE 9:**
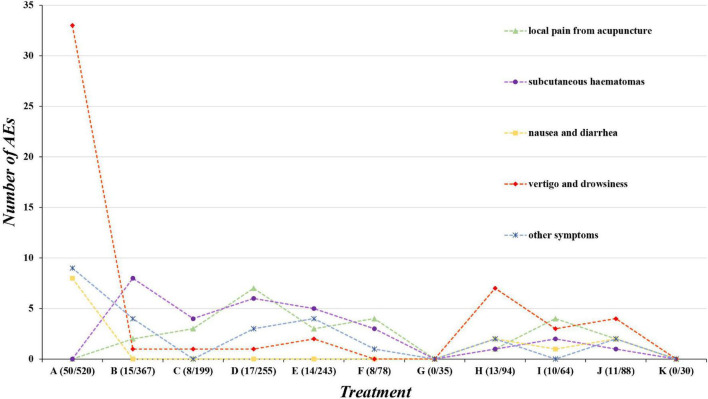
Adverse events. A = antiepileptics; B = manual acupuncture; C = electroacupuncture; D = fire needling; E = pricking and cupping; F = Fu’s acupuncture; G = medicated thread moxibustion; H = electroacupuncture plus antiepileptics; I = fire needling plus antiepileptics; J = pricking and cupping plus antiepileptics; K = acupoint catgut embedding plus antiepileptics.

Of the 29 studies, 16 reported AEs (Tian, 2013; Zhang, 2013, 2015; Sheng et al., 2014; Lei, 2015; Hong, 2016; Wu and Zhao, 2016; Yang and Pan, 2016; Xie et al., 2018; Xu, 2018; Zhou and Huang, 2018; Zhong, 2019; Pang, 2020; Zhao et al., 2020; Ding et al., 2021; Zhang et al., 2021), while the remaining studies did not describe AEs. We divided the AEs into five categories: local pain from acupuncture, subcutaneous hematoma, nausea and diarrhea, vertigo, drowsiness, and others, such as influenza, during treatment. Pricking and cupping plus antiepileptics and EA plus antiepileptics were associated with the highest number of AEs. Fire needling plus antiepileptics had the highest incidence of AEs (14.06%). Additionally, the highest incidences of AEs were vertigo and drowsiness, subcutaneous hematoma, and local pain from acupuncture (35.62, 20.55, and 17.81%, respectively). Briefly, acupuncture and related therapies are associated with an increased incidence of subcutaneous hematoma and local pain from acupuncture pain, whereas antiepileptics and their combined therapies are more associated with reports of vertigo and drowsiness. No severe AEs occurred in any study (Figure 9).

### 3.6. Publication bias

**FIGURE 10:**
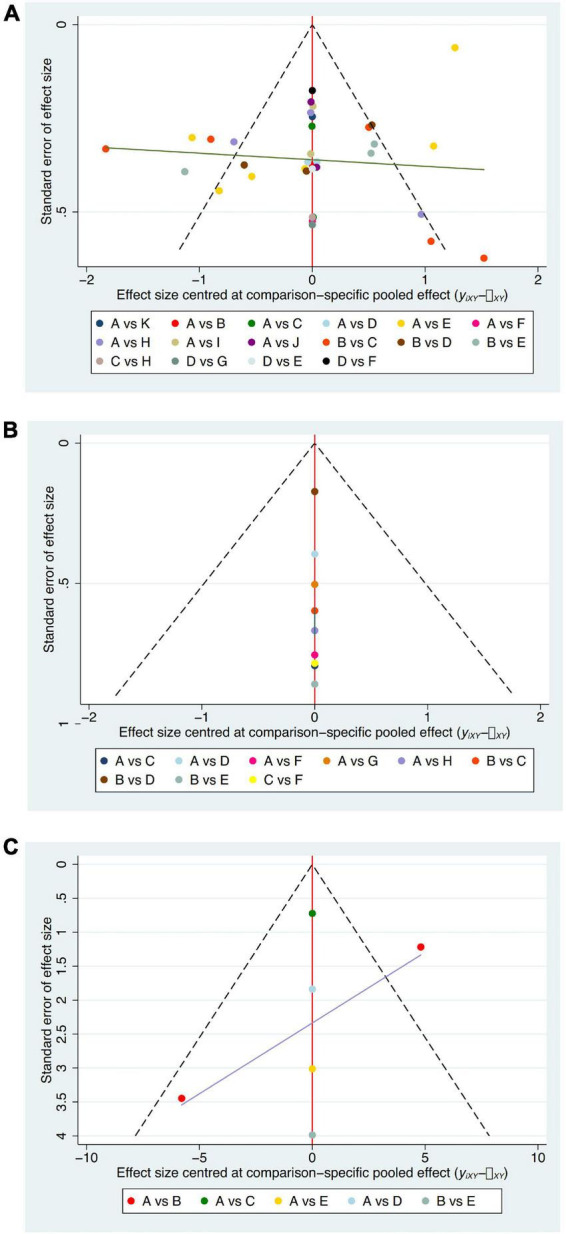
Adjusted funnel plot. **(A)** VAS; **(B)** PSQI; **(C)** SDS; A = antiepileptics; B = manual acupuncture; C = electroacupuncture; D = fire needling; E = pricking and cupping; F = Fu’s acupuncture; G = medicated thread moxibustion; H = electroacupuncture plus antiepileptics; I = fire needling plus antiepileptics; J = pricking and cupping plus antiepileptics; K = acupoint catgut embedding plus antiepileptics.

Publication bias was examined using an adjusted funnel plot (Figure 10); most points were evenly distributed on both sides of the midline and concentrated in the middle region. The reasonable assumption is that the sample size of most included studies was moderate, and therefore the degree of bias in these studies was low. Nevertheless, several points were beyond the two dotted lines, indicating heterogeneity among studies.

## 4. Discussion

There is currently no disease-relieving therapy for PHN; therefore, treatment should focus on controlling symptoms. Besides the physical pain symptoms of PHN patients, the negative emotions caused by pain often lead to symptoms like depression and anxiety (Johnson and Rice, 2014). Most clinical attention with existing therapies is often paid to pain treatment but ignores its negative emotional management, which is difficult to safely and effectively address with state-of-the-art treatments (Boersma et al., 2019). In addition, a previous study confirmed that spontaneous PHN pain has a specific pattern of brain activity, and the regions involved in emotion best reflect the changes in pain after treatment (Geha et al., 2007). Therefore, improved therapeutic approaches are needed to affect patients’ emotional states in the context of pain management therapy.

This is the first NMA for acupuncture and related therapies for PHN. Although evidence has shown that acupuncture is effective in treating PHN (Pei et al., 2019; Zhou et al., 2021), methods vary, and techniques with poor efficacy affect the patient’s condition and waste medical resources. Accordingly, we attempted to identify the optimal PHN acupuncture treatment. The original protocol divided the intervention into four categories: acupuncture techniques alone, sham placebo acupuncture, effective pharmacotherapy, and single acupuncture plus effective pharmacotherapy. Unfortunately, studies regarding effective drug treatments related to acupuncture did not include criteria. Thus, the only pharmacotherapy included in this study, was antiepileptics. Comparisons between acupuncture and antiepileptics are meaningful because antiepileptics are the recommended first-line treatment for PHN with fewer side effects than other effective drugs. No suitable sham-placebo acupuncture was included in this study, and the placebo effect of acupuncture in PHN was not demonstrated. Moreover, because the PHN diagnostic criteria are not standardized, we included two sets of representative PHN criteria in English and Chinese to include a broader collection of literature. Different diagnostic criteria may lead to different clinical implications, but both criteria are generally based on persistent chronic neuropathic pain after the rash healed. However, it also may increase the heterogeneity, affecting the strength of the evidence. Subsequent studies should unify PHN diagnostic criteria, update the expert consensus to facilitate comparisons, and improve the quality of evidence.

Our quality assessment of the 29 RCTs on acupuncture and related therapies for PHN revealed that five RCTs were at high risk of bias. Allocation concealment resulted in the highest risk, which may have led to selection bias. We did not consider studies that did not blind researchers high risk because of the unique clinical features of acupuncture techniques. Unlike clinical drug studies, researchers cannot properly use blinding in acupuncture. Therefore, further high-quality and multicenter clinical trials are required to improve the quality of the evidence.

A total of 1,973 patients were included in this study. VAS, SDS, and PSQI scores and adverse events were evaluated for 10 acupuncture and related techniques and compared with those of antiepileptics for PHN, including manual acupuncture, EA, fire needling, pricking and cupping, Fu’s subcutaneous needling, medicated thread moxibustion, EA plus antiepileptics, fire needling plus antiepileptics, pricking and cupping plus antiepileptics, and acupoint catgut embedding plus antiepileptics. Although 25 studies reported the clinical response rate, it was not included in the comprehensive analysis because most studies were based on different self-designed methods of measuring efficacy. Guidelines or expert consensus for acupuncture treatment of PHN should use unified efficacy criteria to standardize outcome reporting and help with evidence comparisons.

Our NMA results generally followed those of the pairwise meta-analysis when comparing antiepileptics with all types of acupuncture. These treatments reduced VAS scores more effectively than antiepileptics. Regarding PSQI score reduction, only EA plus antiepileptics was more effective than antiepileptics, while the reduction was not significantly different for the other six acupuncture-related therapies. Notably, there was only one study for each intervention. No significant difference among the four acupuncture-related treatments was observed for SDS score reduction compared with antiepileptics. Similarly, only four studies included the SDS outcomes. Some comparisons in the pairwise meta-analysis results were heterogeneous, mainly in EA and pricking and cupping interventions. However, due to our strict inclusion and exclusion criteria, we believe that this heterogeneity does not come from the study designs but rather from clinical heterogeneity. Because of the particularity of acupuncture therapy, it is difficult for acupuncturists to accurately use fixed acupuncture depth and stimulation intensity, which we believe is the main source of clinical heterogeneity. Future studies should adopt unified and standardized techniques, reducing the heterogeneity of acupuncture clinical research. Therefore, these results should be interpreted with caution.

Our ranking probability results suggested that pricking and cupping plus antiepileptics are the most effective pain relief, followed by EA plus antiepileptics; EA plus antiepileptics seems to be most effective in PSQI and SDS score reduction. Additionally, the other eight methods have moderate pain relief effects, although it is difficult to determine which single acupuncture treatment is the best, considering the complexity of the results. Nevertheless, pricking and cupping plus antiepileptics and EA plus antiepileptics have better effects on PHN. We can assume that antiepileptics combined with acupuncture techniques enhance analgesic effects. Currently, which parts play a key role, or which interact to amplify efficacy is unknown. Indeed, this potential hypothesis must be confirmed by basic follow-up research. This provides a new direction for clinical treatment of PHN and, perhaps, a novel approach for treating other types of chronic neuropathic pain using acupuncture.

Modern studies have shown that the analgesic mechanism of EA stimulation with different frequencies differs. It is essential to select the appropriate parameters of EA to optimize the therapeutic effect of EA. Some of the included studies did not detail the frequency parameters used by EA, but most of the reported RCTs with EA used dilatational waves. In rat experiments, 2-Hz electrical stimulation at acupoints can cause the release of high levels of enkephalins and endorphins in the brain and spinal cord. In contrast, 100-Hz electrical stimulation can cause the release of a high level of dynorphin in the spinal cord. Both have analgesic effects, but each has specific characteristics. If a dilatational wave alternating between 2 and 100 Hz is used, the above three peptides can be released simultaneously and play a synergistic analgesic effect (Han, 2011). However, there is still controversy regarding the parameters of EA (Sun et al., 2022). More research is needed on which EA parameters are most effective in treating PHN. In addition, we also summarized the acupoints for treating PHN: the Ashi point was the most frequently used acupoint, followed by the Jiaji point (EX-B2). Ashi acupoints are located in the most painful area of the skin, which can be understood as points located in the sensitized state in the pathological state, and acupoint sensitization can be regarded as a modern supplement to Ashi acupoints. Jiaji acupoints (EX-B2) are located 0.5 cun bilateral to the posterior midline in the dorsal thoracic and lumbar region, ranging from the first thoracic vertebra to the fifth lumbar vertebra. Stimulation can inhibit the expression of pro-inflammatory cytokines and Nogo-NgR signaling pathway, promote nerve growth, and regulate related nerves and organs (Sun et al., 2022). This perspective explains why these two acupoints were used most frequently. However, the research on the complex mechanism of the acupoint effect and the difference in curative impact caused by different acupoint selections is still incomplete and needs further exploration.

The mechanism of acupuncture analgesia for PHN has not yet been clarified despite advances in neurobiology and pain signal transmission. Acupuncture analgesia in PHN is a complex network regulation mechanism involving the entire nervous system from the periphery to the central, and many biologically active substances are involved in acupuncture’s inhibitory effect on PHN. Identifying the mechanism of acupuncture in the treatment of PHN from a pathophysiological perspective is essential. Animal models are used to explain the curative effect of acupuncture on PHN, although there is a dearth of published studies.

Therefore, we summarized current mainstream acupuncture analgesic mechanisms into seven aspects, to provide ideas and a basis for further exploration of acupuncture analgesia in PHN treatment. (1) Acupuncture modulates the interaction between nociceptive receptors and the inflammatory immune response, inhibiting the release of inflammatory cytokines, lipids, prostaglandin E2, 5-hydroxytryptamine, histamine, and nerve growth factor by immune cells, inhibiting peripheral receptor nociceptor sensitization and transient receptor potential cation channel subfamily V member 1 phosphorylation, alleviating peripheral sensitization resulting in pain relief (Zhang et al., 2014; Zhang Y. et al., 2020; Hsu et al., 2019). (2) Sodium channels are involved in the overexcitation of sensory neurons leading to neuropathic pain, and acupuncture can change sodium ion channel expression to produce analgesic effects (Lu et al., 2016; Yen et al., 2018; Li et al., 2020). (3) Although PHN is caused by peripheral nervous system injury, neuropathic pain is maintained by central sensitization, that is, plasticity changes in the central nervous system (Meacham et al., 2017). Acupuncture can inhibit central sensitization and the formation of synaptic plasticity, achieving analgesic effects (Ji et al., 2018; Lai et al., 2019; Qiao et al., 2019). (4) Spinal glial cells (microglia and astrocytes) are involved in inflammatory responses and neuropathic pain, and glial cells—especially microglia—play key roles in the pathogenesis of neuropathic pain. Acupuncture reduces pain by inhibiting this activation (Lin et al., 2016; Wang J. Y. et al., 2018). (5) Acupuncture can regulate the entire brain area network of pain stimulation by adjusting the interaction between various brain functional areas and neurotransmitters, producing analgesic effects (Yuan et al., 2018; Zhao et al., 2018). (6) Acupuncture exerts analgesic effects by mediating mitogen-activated protein kinase (MAPK) signaling pathways, including the spinal cord p38/MAPK (Fang et al., 2013; Liang et al., 2016), extracellular-signal-regulated kinase (Choi et al., 2012; Park et al., 2014), c-Jun N-terminal kinase (Lee et al., 2013), and nuclear factor kappa-B pathways (Park et al., 2002). (7) Acupuncture exerts analgesic effects through epigenetic regulation of genes in tissues and cells, including DNA methylation (Jang et al., 2021), histone modification (Lai et al., 2012), and microRNA regulation (Liu and Wu, 2017; Zou et al., 2019). These theories have provided a theoretical basis for evaluating the effectiveness of acupuncture in PHN treatment and have laid the foundation for follow-up research.

We analyzed 16 RCTs that reported AEs—summarized into five aspects: local pain from acupuncture, subcutaneous hematoma, nausea and diarrhea, vertigo and drowsiness, and other symptoms. Vertigo and drowsiness, subcutaneous hematoma, and local pain from acupuncture were the most common AEs. Antiepileptics- (including combined acupuncture treatments) mainly relate to vertigo and drowsiness, and acupuncture-related techniques mainly caused subcutaneous hematoma and local pain. Therefore, we do not recommend antiepileptics for patients with PHN comorbid with neurological diseases or older people, although combined therapy is better than acupuncture alone for pain relief. Patients with renal deficiency should also avoid antiepileptics due to drug contraindications. Patients should be informed of the possibility of mild pain and subcutaneous hematoma before acupuncture-related treatment; if they are intolerant, acupuncture should also be avoided. Concisely, acupuncture is relatively safe compared to antiepileptics because acupuncture pain is mild and transient.

In conclusion, for the sole purpose of relieving pain, the clinician should choose pricking and cupping plus antiepileptics, while PHN patients with negative emotions and insomnia should use electroacupuncture plus antiepileptics. Nevertheless, these two treatments both had the most AEs, combining the side effects of both antiepileptic and acupuncture treatments. This presents a thought-provoking approach to personalized precision therapy. In future clinical research, patients should be divided according to degrees of pain; for example, combined treatment is recommended for severe and moderate pain, and acupuncture alone is recommended for mild pain. This indicates the future direction of combined acupuncture and Western medicine treatments for PHN and may inspire other combined acupuncture and Western medicine treatment programs. However, this is currently only a hypothesis that requires further testing.

### 4.1. Strengths

First, this review was strictly conducted using PRISMA and PRISMA-NMA guidelines. Second, to ensure adequate retrieval, we searched eight Chinese and English electronic databases and searched two clinical trial registration platforms, previous meta-analyses, gray literature, and reference lists of selected studies. Third, because RCTs are the gold standard for clinical trials, we excluded unqualified RCTs or non-RCTs, which might have affected our outcomes. Fourth, we tried to divide Western drugs by category to improve the quality of evidence comparison, although only one drug met the criteria. Fifth, we used Bayesian multiple-treatment NMA to acquire more accurate estimates than the frequentist framework method. Finally, the network results of our study are reliable because the *P-*values of the inconsistent node-splitting analysis were all greater than 0.05, and all curves of the diagnostic convergence graph approach 1.

### 4.2. Limitations

First, high-quality RCTs were rare, and the sample sizes in most studies were small; therefore, this finding’s power is limited. Second, we used two diagnostic criteria, which increased heterogeneity, affecting the power of evidence. Third, the long-term efficacy of the interventions involved in this study has not been verified. Fourth, all included studies were performed in China; thus, language bias is possible. Fifth, there is no evidence to guide the choice of optimal acupoints, treatment frequency, or duration in this study. Finally, the study lacked clinical response rate and quality-of-life indicators.

### 4.3. Future prospects

In the future, basic studies should explore the potential mechanism of acupuncture, fully utilizing modern neurobiological technology to explore the complex network mechanism of acupuncture treatment for PHN. Second, basic research should focus on more than just animal models, as research on human biochemical indicators is lacking. Multiomics technology can conduct *in vitro* experiments to detect changes in related metabolites and signal pathway molecules at all levels in patients with PHN before and after acupuncture, further clarifying the mechanism of action and exploring the potential value of acupuncture therapy for PHN. Third, subsequent studies should unify the diagnostic criteria of PHN and modify the expert consensus to facilitate the comparison, and improve the quality, of evidence. Fourth, existing studies lack quality-of-life outcomes and unified evaluation criteria for the clinical response rates. Follow-up studies should adopt unified clinical efficacy evaluation criteria and gradually establish a set of homogeneous outcome indicators directly related to patient interests. Fifth, long-term follow-up and acupuncture placebo groups should be in future studies to clarify thelong-term efficacy and placebo effect of acupuncture. Sixth, there are no comparative studies on the long-term efficacy of different acupuncture techniques for chronic pain; therefore a relevant NMA should be conducted to find the best acupuncture-related technique. Seventh, given the mixed nature and complexity of acupuncture techniques and antiepileptics for PHN, further research should focus on classifying the degree of pain in patients with PHN into discrete subtypes, ultimately providing the basis for more personalized clinical treatment schemes. Eighth, the clinical treatment of acupuncture and moxibustion should adopt unified and standardized techniques, such as the same basic points, acupuncture depth, acupuncture frequency, and treatment course. This is not contradictory to individualized treatment, although it is more convenient for evidence comparisons, thus reducing the heterogeneity of acupuncture clinical research and improving the quality of evidence. Finally, most Chinese studies on acupuncture do not share original research data. Data sharing and transparency in clinical studies should be supported to facilitate data mining and reusing clinical data for acupuncture therapy.

### 4.4. Comparison

Coincidentally, one study on a similar topic (Wang et al., 2022) was published just a few days ago. Therefore, we decided to compare our analysis with theirs. First, NMA is mainly based on two theoretical frameworks: frequency and Bayesian. We used R software to conduct NMA based on the Bayesian framework, while Wang et al. (2022) used STATA software to conduct a frequency-based NMA. Notably, the frequency method estimates the maximum likelihood function by constantly iterating during parameter estimation, and the result is more likely to be biased. The Bayesian approach does not have this problem, and the estimation value is more accurate (Hutton et al., 2015). Second, we not only analyzed the outcome indicators of pain reduction and AEs but also conducted NMA on improvement in pain-related outcomes (sleep quality and depression), whereas Wang et al. (2022) only analyzed pain reduction and AEs. Third, our study search strategy was more comprehensive, searching not only entire electronic databases but also clinical trial registries and gray literature, ensuring a comprehesive literature search’. In addition, we also recorded the exact reasons for the elimination of each study (Supplementary Appendix 2). Fourth, the method for measuring clinical response rates is based on two main categories: (1) objective measures based on the percentage reduction in pain scores and (2) subjective measures based on improvement in patients’ self-perceived pain. Therefore, our study could not comprehensively analyze of this outcome. Although Wang et al. (2022) analyzed the efficiency rate outcome, they used two different efficacy evaluation criteria. There is heterogeneity in this method, reducing the evidence’s power for this outcome. Notably, we pointed out the solution to this problem in the discussion. Fifth, we evaluated the similarity, consistency, and convergence of the results among the included RCTs to ensure the feasibility of NMA and the stability of its results, while Wang et al. (2022) did not. Sixth, we believe that acupoint injection is a combination of needle and medicine, a unique injection therapy in which drugs inject into specific acupoints through a syringe. According to our investigation, the types and dosages of drugs used for acupoint injection in RCTs are also inconsistent. Therefore, we did not include acupoint injection technique as opposed to Wang et al. (2022), and we believe that the significance of this treatment is limited. Seventh, we made a refined classification of first-line PHN drugs, although our study included only antiepileptics. Nonetheless, the control group in the study by Wang et al. (2022) combined different types of drugs into the same Western medicine category, reducing the strength of the evidence. Eighth, we discussed and summarized the analgesic mechanism of acupuncture in the treatment of PHN, as well as deficiencies and prospects of study on acupuncture-related therapies, to clarify its analgesic mechanism and provide ideas for subsequent research.

In addition, we compared the protocol of Bian et al. (2022) with our study, although its formal study has yet to be accomplished. First, the treatment was limited to a single acupuncture technique in our study, while Bian et al. (2022) allowed multiple acupuncture treatments to be used in combination. In clinical practice, physicians are more inclined to use a single, effective acupuncture treatment, which can reduce patients’ cost burden and the fear and pain of acupuncture stimulation. Therefore, the results of their protocol may lead to confusion regarding the clinician’s selection. Furthermore, due to the mixing and complexity of multiple acupuncture combinations, Bian et al. (2022) are unable to specify which acupuncture technique plays the most important role, which cannot provide a reference for subsequent clinical and basic research. Second, we made a refined classification of first-line PHN drugs, although our study included only antiepileptics. Nonetheless, the control group for Bian et al. (2022) only selects conventional drugs vaguely, without limiting the use of recognized first-line PHN treatment drugs and without precise classification of drugs, which reduces the strength of the quality of evidence for comparison. Finally, we discussed and summarized the analgesic mechanism of acupuncture in the treatment of PHN as well as deficiencies and prospects of study on acupuncture-related therapies to clarify its analgesic mechanism and provide ideas for subsequent research.

## 5. Conclusion

Based on the evidence from our analysis, acupuncture-related therapies may be potential treatment options for PHN and are relatively safe with only mild side effects. Pricking and cupping plus antiepileptics is the most effective acupuncture-related technique for PHN pain reduction, followed by EA plus antiepileptics. Moreover, EA plus antiepileptics is the best acupuncture-related technique for improving PHN-related insomnia and depression symptoms. Nevertheless, owing to the limitations of this study, these conclusions should be cautiously interpreted, and future high-quality studies are needed.

## Data availability statement

The original contributions presented in this study are included in this article/Supplementary material, further inquiries can be directed to the corresponding authors.

## Author contributions

YC: conception and design and drafting and writing. XYZ, QL, DW, and JZ: retrieval and screening of studies and extraction of data. XXZ and QH: evaluation of the methodological quality of included studies. RY, SX, and DZ: statistical analysis. XM and SZ: revision of chart. HY and ZS: review and revision. All authors contributed to the article and approved the submitted version.

## Footnotes

1
https://trialsearch.who.int

2
https://www.chictr.org.cn

3
http://www.opengrey.eu
