# Supplementary material for: Efficacy of different acupuncture therapies on postherpetic neuralgia: A Bayesian network meta-analysis
Source: Front Neurosci. 2023 Jan 10;16:1056102. doi: 10.3389/fnins.2022.1056102 (PMC9871906; doi:10.3389/fnins.2022.1056102)
Supplement: Supplementary file 1 [file Data_Sheet_1.DOCX]

Lewith, GT, 1983

Rutgers MJ, 1988

Li MIN, 2000

Lei ZJ, 2001

Liu Q, 2002

Li M, 2002

Wu JQ, 2002

Yu CY, 2002

Zuo QP, 2003

Zhao XM, 2003

Tan QW, 2004

Zhu TB, 2004

Cheng XQ, 2004

Liu B, 2004

Liu JW, 2004

Gao HY, 2005

FAN Jin, 2005

Zhao HR, 2005

Yu J, 2006

Yin Y, 2006

Zhao GL, 2006

Qin XZ, 2006

Fang WD, 2006

Cheng JJ, 2006

Wang J, 2006

Shi YC, 2007 Malcolm R Ing,2007

Cheng ZJ, 2007

Chen JX, 2007

Liu JW, 2007

Li YK, 2007

Wu J, 2007

Lin GH, 2007

Zhao XX, 2007

Yu WX, 2007

Lin GC, 2007

Ma JY, 2007

Wu RF, 2007

Chen YZ, 2008

Zhou ZK, 2008

Huo MX, 2008

Zhong J, 2008

Sun X, 2008

Mao M, 2008

Shi ZH, 2008

**Exclusion studies and reasons (****total records 224, number on this page = 45)**

**Reasons**

**Exclusion studies**

Study without clear criteria for efficacy.

Full text cannot be obtained.

Study without necessary outcomes.

Cupping and manual acupuncture were used in combination.

Cupping and manual acupuncture were used in combination.

Acupuncture and brachial plexus closure were used in combination.

Study without clear diagnostic criteria.

Study without clear random grouping method.

Study without clear random grouping method.

Full text cannot be obtained.

Study was randomly assigned according to the order of visit.

Acupuncture plus bloodletting puncture and cupping were used in combination.

Full text cannot be obtained.

Study without clear diagnostic criteria.

Study without clear diagnostic criteria.

Study without clear diagnostic criteria.

Study without clear diagnostic criteria.

Study without clear random grouping method.

Manual acupuncture and moxibustion therapy

Study without clear random grouping method.

Manual acupuncture and cupping were used in combination.

Participant didn’t meet the PHN diagnostic criteria.

Study without clear diagnostic criteria.

Study was randomly assigned according to the order of visit.

Study without clear random grouping method.

Study without clear random grouping method.

The results are shown as a percentage and the conversion error is large.

Cupping and blooding plus manual acupuncture were used in combination.

Study without clear diagnostic criteria.

Cupping and blooding plus manual acupuncture were used in combination.

Fire needle and acupuncture were used in combination.

Full text cannot be obtained.

Study without clear diagnostic criteria.

Study without baseline data.

Study without clear random grouping method.

Study without clear random grouping method.

Manual acupuncture and carbamazepine were used in combination.

Study without clear random grouping method.

Bloodletting and acupuncture were used in combination.

Study of acupuncture manipulation.

Study without clear diagnostic criteria.

Study without clear diagnostic criteria.

Study without clear random grouping method.

Manual acupuncture and fire needling were used in combination.

Manual acupuncture and electroacupuncture were used in combination.

**Continued (number** **on this page = 45)**

**Reasons**

**Exclusion studies**

Study without clear random grouping method.

Study without clear diagnostic criteria.

Study without clear diagnostic criteria.

Study without clear random grouping method.

Study without clear random grouping method.

Cupping and blooding and moxibustion were used in combination.

Study without clear random grouping method.

Treatment group used acupoint injection.

Study without clear random grouping method.

Electroacupuncture and fentanyl patch were used in combination.

Study without clear diagnostic criteria.

Study without clear random grouping method.

Manual acupuncture and TDP were used in combination.

Fire needle and acupuncture were used in combination.

Manual acupuncture and TDP plus cupping were used in combination.

Study without clear diagnostic criteria.

Manual acupuncture and TENS were used in combination.

Manual acupuncture and bloodletting were used in combination.

Manual acupuncture and TDP were used in combination.

Acupoint catgut embedding and cupping were used in combination.

Electroacupuncture and carbamazepine used in combination

Treatment group used TENS.

Full text cannot be obtained.

Study of acupuncture manipulation.

Study without clear diagnostic criteria.

Manual acupuncture and bloodletting were used in combination.

Study without clear diagnostic criteria.

Study without necessary outcomes.

Study without clear diagnostic criteria.

Full text cannot be obtained.

Treatment for the control group was not described.

Bloodletting and encircling needling were used in combination.

Study without clear random grouping method.

Study without clear random grouping method.

Study without baseline data evaluation.

Study without clear random grouping method.

Study without clear diagnostic criteria.

Full text cannot be obtained.

Same data as Huang JM, 2011.

Study was randomly assigned according to the order of visit.

Study without clear random grouping method.

Study without clear diagnostic criteria.

Study without baseline data evaluation.

Full text cannot be obtained.

Study was randomly assigned according to the order of visit.

Wang SL, 2008

Tian J, 2008

Chen WS, 2008

Wang XC, 2008

Dong YX, 2008

Pang SF, 2009

Liu J, 2009

Li CY, 2009

Zhang SJ, 2009

Zhang DQ, 2009

Duan Q, 2009

Min XJ, 2009

Chen M, 2009

Chen HP, 2009

Li JH, 2010

Liang MA, 2010

Niu SM, 2010

Fang XJ, 2010

Zhao JX, 2010

Yu XF, 2010

Zou R, 2010

Barbarisi M, 2011

Isrctn, 2011

Zhou ZK, 2011

Li CY, 2011

Fang XJ, 2011

Xu ZJ, 2011

Lin C, 2011

Tian Q, 2011

Tian JZ, 2011

Guo L, 2011

Zhao RQ, 2011

Li L, 2011

Lin C, 2011

Chen XX, 2011

Chen CX, 2011

Cai ZL, 2011

Zhu YJ, 2011

Li L, 2012

Cheng JJ, 2012

Zhao XB, 2012

Bai DS, 2012

You XB, 2012

Pei GD, 2012

Xie YH, 2012

**Continued (number on this page = 45)**

Li JJ, 2012

Wu ZJ, 2012

Fang XY, 2013

Liang XS, 2013

Chen LJ, 2013

Chen ML, 2013

Tong B, 2013

Ye HS, 2013

Zhuang X, 2013

Wang WJ, 2013

Hu CL, 2013

Gong J, 2013

Ma K, 2013

Xu ZJ, 2013

Li LP, 2013

Xu H, 2013

Yang J, 2013

Bian F, 2013

Zhong YL, 2013

Ma XP, 2013

Wu XT, 2013

He YW, 2013

Tian H, 2013

Li JJ, 2014

Huang SL, 2014

Lu MC, 2014

Guo LH, 2014

Tong B, 2014

Meng L, 2014

Zheng YJ, 2014

Pang KY, 2014

Wang H, 2014

Chen YK, 2014

Liu B, 2015

Feng QT, 2015

Zhang XQ, 2015

Wang JM, 2015

He QT, 2015

Wu ML, 2015

Liu M, 2015

Li J, 2015

Li C, 2015

Dai XX, 2015

Ji XX, 2016

Wu JX, 2016

**Reasons**

**Exclusion studies**

Study without clear diagnostic criteria.

Study without clear random grouping method.

The efficacy criteria are inconsistent.

Study without clear random grouping method.

Study without clear criteria for efficacy.

Study without clear random grouping method.

Study without baseline data evaluation.

Study without clear diagnostic criteria.

Manual acupuncture and antiviral were used in combination.

Study without clear random grouping method.

Study without clear random grouping method.

Study without clear diagnostic criteria.

Treatment group used acupotomology therapy.

Full text cannot be obtained.

Study without clear random grouping method.

Full text cannot be obtained.

Fire needle and manual acupuncture were used in combination.

Study without necessary outcomes.

Moxibustion and bloodletting were used in combination.

Full text cannot be obtained.

Study was randomly assigned according to the order of visit.

Treatment group used TENS.

Study was randomly assigned according to the order of visit.

Study without clear random grouping method.

Study without clear random grouping method.

Study without clear diagnostic criteria.

Manual acupuncture and laser plus needle knife were used in combination.

Confused outcome indicators.

Study without baseline data evaluation.

Study without clear diagnostic criteria.

Study without clear random grouping method.

Study without clear random grouping method.

Study without clear random grouping method.

Study without clear diagnostic criteria.

Study without clear diagnostic criteria.

Full text cannot be obtained.

Fire needle and cupping were used in combination.

Study without clear diagnostic criteria.

Full text cannot be obtained.

Study was randomly assigned according to the order of visit.

Study without clear random grouping method.

Study without clear random grouping method.

Fu’s acupuncture and carbamazepine were used in combination.

Study without clear random grouping method.

Manual acupuncture and acupoint injection were used in combination.

**Reasons**

**Exclusion studies**

**Continued (****number on this page = 45)**

Huang WY

Ji J, 2016

Zhang C, 2016

Li YJ, 2016

Zeng F, 2016

Sun LX, 2016

Xu QE, 2017

He XH, 2017

Liu MH, 2017

Zhang XX, 2017

Bian F, 2017

Hou JT, 2017

Xia YZ, 2018

Wu CY, 2018

Xia YZ, 2018

Lin SY, 2018

Wang SZ, 2018

Wang SM, 2018

Chen LJ, 2018

Zhang Y, 2018

Li WP, 2018

Tian F, 2018

Zheng YF, 2018

Chen XF, 2018

Gong YY, 2018

Bai EH, 2018

Nct, 2019

Liu FN, 2019

Zhang EY, 2019

Li WQ, 2019

Qin L, 2019

Zheng DF, 2019

Jin ZX, 2019

Gu GQ, 2019

Nct, 2019

Li XJ, 2019

He, DD, 2019

Ni SL, 2019

Shao NL, 2019

Ding LW, 2019

Chen Y, 2019

Liu QQ, 2019

Chen Y, 2019

Marwa Eid, 2020

Ren YW, 2020

Study without clear diagnostic criteria.

Same data as Lei, 2015.

No specific outcomes data were available.

Treatment group used TENS.

Study without clear diagnostic criteria.

Study without clear random grouping method.

Manual acupuncture and bloodletting were used in combination.

Bloodletting and warm needle were used in combination.

Fire needle and cupping were used in combination.

Full text cannot be obtained.

Duplicated publish.

Study without clear random grouping method.

Duplicated publish.

Manual acupuncture and moxibustion were used in combination.

Study without necessary outcomes.

Acupoint catgut embedding and Fu’s subcutaneous needling were combined.

Study without clear random grouping method.

Study without clear random grouping method.

Fu’s acupuncture and manual acupuncture were used in combination.

Study without clear diagnostic criteria.

Study without clear diagnostic criteria.

Manual acupuncture and moxibustion were used in combination.

Study without clear diagnostic criteria.

Fire needle and encircling needling were used in combination.

Full text cannot be obtained.

Study without clear random grouping method.

No results posted.

Study without clear diagnostic criteria.

Moxibustion and manual acupuncture were used in combination.

Study without clear diagnostic criteria.

Full text cannot be obtained.

Fire needle and encircling needling were used in combination.

Study without clear diagnostic criteria.

Study without clear diagnostic criteria.

No results posted.

Study was randomly assigned according to the order of visit.

Study without clear random grouping method.

Fire needling and polarized light were used in combination.

Study without clear random grouping method.

Study without clear diagnostic criteria.

Study without clear random grouping method.

Fire needling plus external application of Traditional Chinese medicine.

Study without clear random grouping method.

Non outcomes.

Study without clear random grouping method.

Liu L, 2020

Fei EDABLZ, 2020

Wang L, 2020

Wang J, 2020

Gu Y, 2020

Tang PW, 2020

ChiCtr, 2020

Kang YJ, 2020

Yuan L, 2020

Deng WY, 2020

Zhang SY, 2020

Zhang YP, 2020

Zhang L, 2020

Lu L, 2020

Guo JZ, 2021

Deng MN, 2021

Li JF, 2021

Li LM, 2021

Zheng GJ, 2021

Zhou WH, 2021

Tian ZX, 2021

Zheng XC, 2021

Cui WJ, 2021

Li YW, 2021

Zhang YN, 2021

Zou WL, 2021

Xing XY, 2021

Sollie M, 2022

[Jingjing Lai](https://www.webofscience.com/wos/alldb/general-summary?queryJson=%5B%7B%22rowField%22:%22AU%22,%22rowText%22:%22Jingjing,%20Lai%22%7D%5D&eventMode=oneClickSearch), 2022

Peng WQ, 2022

Yin H, 2022

Chen N, 2022

Hu YC, 2022

Liao YM, 2022

Zhang YZ, 2022

Wang Y, 2022

Zhang HY, 2022

Liu SF, 2022

Liu YB, 2022

Wang XM, 2022

Wang SS, 2022

Li ZW, 2022

Cebeci D

[Huang YF](https://www.webofscience.com/wos/alldb/general-summary?queryJson=%5B%7B%22rowField%22:%22AU%22,%22rowText%22:%22Huang,%20YF%22%7D%5D&eventMode=oneClickSearch), 2022

Study without clear diagnostic criteria.

Study without clear random grouping method.

Electroacupuncture plus external application of Traditional Chinese medicine.

Study without clear random grouping method.

Study without eligible outcome measures.

Study without clear diagnostic criteria.

Full text cannot be obtained.

Study without clear diagnostic criteria.

Study without clear random grouping method.

The studyx adopted Chinese medicine nursing.

Study without clear diagnostic criteria.

Study without clear random grouping method.

Study without clear diagnostic criteria.

Electroacupuncture and fire needle were used in combination.

Study without clear random grouping method.

Manual acupuncture and Chinese medicines were used in combination.

Study without clear random grouping method.

Treatment group used acupoint injection.

Manual acupuncture and Chinese medicines were used in combination.

Study without clear random grouping method.

The treatment group used interferon.

Manual acupuncture and blooding were used in combination.

Study without clear random grouping method.

Manual acupuncture combined with valaciclovir.

Study without clear random grouping method.

Electroacupuncture and TENS were used in combination.

Study without clear random grouping method.

Dry acupuncture and sham acupuncture were compared, but NMA could not be performed without studies of the same intervention.

Zhuang medicine lotus acupuncture and cupping were used in combination.

Electroacupuncture treatment and conventional therapy were used in combination.

Study compared the differences in electroacupuncture frequency.

WIRA, pregabalin, and fire needle were used in combination.

Manual acupuncture and plum flower needle were used in combination.

Intradermal injection, manual acupuncture, moxibustion were used in combination.

Catgut embedding plus autotomy therapy were used in combination.

Moxibustion with seed-sized moxa cone and manual acupuncture were used in combination.

Study compared the differences in acupuncture manipulation.

Acupuncture plus injection therapy.

Acupuncture plus moxibustion.

Study without clear random grouping method.

Study compared different pressures of cupping.

Non-randomized controlled trial.

Non-randomized controlled trial.

The population are postherpetic neuralgia mixed with myofascial pain syndrome.

**Continued (number** **on this page = 44)**

**Reasons**

**Exclusion studies**
