# Supplementary material for: Efficacy of different acupuncture therapies on postherpetic neuralgia: A Bayesian network meta-analysis
Source: Front Neurosci. 2023 Jan 10;16:1056102. doi: 10.3389/fnins.2022.1056102 (PMC9871906; doi:10.3389/fnins.2022.1056102)
Supplement: Supplementary file 2 [file Data_Sheet_2.DOCX]

R1. 王军.(2006).火针治疗血瘀型带状疱疹后遗神经痛的临床研究(博士学位论文,北京中医药大学).

Wang, J. (2006). Fire needling of blood stasis type of post-herpetic neuralgia clinical research (Ph.D. Dissertation, Beijing university of Chinese Medicine).

R2. 黄瑾明,韩海涛,李婕,陈林,陆璇霖 & 葛春雷.(2011).莲花针拔罐治疗带状疱疹后遗神经痛的疗效观察. 广西中医药(01),31-32.

Huang, J. M., Han, H. T., Li, J. J., Chen, L., Lu, X. L., & Ge, C. L. (2011). Effect of lotus needle cupping therapy on postherpetic neuralgia. Guangxi Traditional Chinese Medicine (01), 31-32.

R3. 陈攀,林辰 & 杨建萍.(2012).药线点灸法与火针法治疗带状疱疹后神经痛的疗效对比分析. 中华中医药杂志(07),1847-1849. doi:CNKI:SUN:BXYY.0.2012-07-037

Chen, P., Lin, C., & Yang, J. P. (2012). Comparative analysis of effects between acupuncture of fire needle and medicated thread moxibustion on postherpetic neuralgia. Chinese Journal of Traditional Chinese Medicine (07), 1847-1849.

R4. 张聪.(2013).围针治疗带状疱疹后神经痛的临床观察及对血清IL-6的影响(硕士学位论文,南京中医药大学).

Zhang, C. (2013). Clinical study on the postherpetic neuralgia with round acupuncture and its effect on IL-6 (Master Dissertation, Nanjing University of Chinese Medicine).

R5. 田浩.(2013).刺血拔罐治疗带状疱疹后遗神经痛的疗效与机制研究(博士学位论文,中国中医科学院).

Tian, H. (2013). Efficacy and mechanism research on treating postherpetic neuralgia by blood-letting puncturing and cupping therapy (Ph.D. Dissertation, China Academy of Traditional Chinese Medicine).

R6. 谢衡辉 & 文娜.(2013).针刺补泻手法用于夹脊穴、阿是穴为主治疗带状疱疹后遗神经痛临床观察. 北京中医药(01),23-27.

Xie, H. H., & Wen, N. (2013). Clinical observation of reinforcing and reducing technique at Jia-ji and Ashi points for post-herpetic neuralgia. Beijing Traditional Chinese Medicine (01), 23-27.

R7. 段丽丽.(2014).夹脊电针治疗带状疱疹后遗肋间神经痛的临床疗效观察(硕士学位论文,黑龙江中医药大学).

Duan, L. L. (2014). The clinical efficacy observation of electroacupuncture Jia-ji for treatment of postherpetic intercostals neuralgia (Master Dissertation, Heilongjiang University of Chinese Medicine).

R8. 盛国滨,段丽丽,蔡玉梅,唐英 & 祝天恩.(2014).夹脊电针治疗带状疱疹后遗肋间神经痛的临床疗效观察. 中医药信息(06),85-86.

Sheng, G. B., Duan, L. L., Cai, Y. M., Tang, Y., & Zhu, T. (2014). Clinical observation of Jia-ji electroacupuncture for postherpetic neuralgia. Information on Traditional Chinese Medicine (06), 85-86.

R9. 孙远征 & 李磊.(2015).电针郄穴结合围刺法治疗带状疱疹后神经痛临床观察. 中国针灸(S1),4-6.

Sun, Y. Z., & Li, L. (2015). Clinical observation of electroacupuncture cleft points combined with peri-acupuncture in the treatment of postherpetic neuralgia. Zhongguo Zhen Jiu (S1), 4-6.

R10. 张玉霞.(2015).不同针刺方法治疗带状疱疹后遗神经痛疗效比较. 上海针灸杂志(07),620-622.

Zhang, Y. X. (2015). Comparative study on therapeutic efficacies of different acupuncture methods for post-herpetic neuralgia. Shanghai journal of acupuncture and moxibustion, 34 (7), 620‐622.

R11. 雷玉婷.(2015).盛氏“祛瘀扶正”针法治疗带状疱疹后神经痛的疗效观察及对血清中5-HT的影响(硕士学位论文,南京中医药大学)

Lei, Y. T. (2015). Clinical study on postherpetic neuralgia by acupuncture treatment of Sheng’s “remove blood stasis and righting” and its effect on serum 5 - HT (Master Dissertation, Nanjing University of Chinese Medicine).

R12. 杨志刚 & 潘春联.(2016).加巴喷丁联合电针疗法治疗带状疱疹后遗神经痛的临床疗效分析. 临床医学工程(01),43-44+47.

Yang, Z. G., & Pan, C. L. (2016). Clinical effect of gabapentin combined with electro-acupuncture therapy for post-herpetic neuralgia. Clinical medical & engineering (01), 43-44+47.

R13. 吴丽丽 & 赵辉.(2016).刺血拔罐治疗带状疱疹后遗神经痛临床观察. 湖北中医杂志(11),57-58.

Wu, L. L., & Zhao, H. (2016). Clinical observation on postherpetic neuralgia with blood pricking and cupping. Hubei Journal of Traditional Chinese Medicine (11), 57-58.

R14. 洪永波.(2016).刺血拔罐治疗头面部带状疱疹后遗神经痛的临床疗效观察.中国中药杂志社.(eds.)中国中药杂志2015/专集：基层医疗机构从业人员科技论文写作培训会议论文集(pp.1424).中国中药杂志编辑部.

Hong, Y. B. (2016). Clinical effect of blood pricking and cupping therapy on postherpetic neuralgia of the head and face. China Journal of Traditional Chinese Medicine.(EDS.). Editorial Department of Chinese Journal of Traditional Chinese Medicine.

R15. 周亚兰 & 黄应杰.(2018).火针点刺心俞、膈俞穴治疗带状疱疹后遗神经痛的临床观察. 天津中医药(01),28-30.

Zhou, Y. L., & Huang, Y. J. (2018). Clinical observation on the treatment of postherpetic neuralgia by acupuncture xin-shu and ge-shu points with fire needle. Tianjin Traditional Chinese Medicine (01), 28-30.

R16. 徐洋洋.(2018).毫火针治疗带状疱疹后神经痛的临床研究(硕士学位论文,湖北中医药大学).

Xu, Y. Y. (2018). Clinical research on treatment of postherpetic neuralgia with filiform-fire needle (Master Dissertation, Hubei University of Chinese Medicine).

R17. 谢意杰,赵敬军,姜淼,袁萍,马余鸿,高清云... & 张莉莉.(2018).围刺联合加巴喷丁治疗带状疱疹后遗神经痛的临床研究. *针灸临床杂志*(09),20-23.

Xie, Z. J., Zhao, J. J., Jiang, M., Yuan, P., Ma, Y. H., Gao, Q. Y., et al., & Zhang, L. L. (2018). Clinical study of surround needling combined with gabapentin treating PHN. Chinese Journal of Acupuncture and Moxibustion (09), 20-23.

R18. 陈世云,李晶晶 & 周鹏.(2018).火针与毫针治疗带状疱疹后遗神经痛的效果对比. 中国医学创新(06),50-53.

Chen, S. Y., Li, J. J., & Zhou, P. (2018). Comparison of the efficacy of fire acupuncture and acupuncture needle in the treatment of postherpetic neuralgia. Chinese Medical Innovation (06), 50-53.

R19. 韩海涛,李美康,李婕,李秀娟,赖菁菁,李伟茜... & 陆璇霖.(2018).壮医莲花针拔罐逐瘀法对PHN患者的镇痛作用及血清IL-18、TNF-α的影响. *广西中医药大学学报*(04),8-11.

Han, H. T., Li, M. K., Li, J., Li, X. J., Lai, J. J., Li, W. Q., et al., & Lu, X. L. (2018). Analgesic effect and serum IL-18 and TNF-α of Zhuang-yi Lotus needle cupping for postherpetic neuralgia. Journal of Guangxi University of Traditional Chinese Medicine (04), 8-11.

R20. 莫媛.(2019).*壮医莲花针拔罐逐瘀法对PHN患者Wnt3a及TNF-α的影响*(硕士学位论文,广西中医药大学).

Mo, Y. (2019). Zhuang Medical Lotus Acupuncture and Cupping Method for PHN patients Effects of Wnt3a and TNF alpha (Master Dissertation, Guangxi University of Chinese Medicine).

R21. 钟衡.(2019).*毫火针治疗带状疱疹后神经痛的疗效观察*(硕士学位论文,广州中医药大学).

Zhong, H. (2019). Clinical observation on the milli-fire-needle in treating postherpetic neuralgia (Master Dissertation, Guangzhou University of Chinese Medicine).

R22. 陈蕾.(2019).*夹脊穴埋线配合普瑞巴林治疗带状疱疹后神经痛的临床疗效观察*(硕士学位论文,黑龙江中医药大学).

Chen, L. (2019). Clinical observation on Jia-ji acupoint catgut embedding combined with pregabalin on treatment for postherpetic neuralgia. (Master Dissertation, Heilongjiang University of Chinese Medicine).

R23. 庞韩星.(2020).*“腕踝毫火针”治疗带状疱疹后遗神经痛的临床疗效观察*(硕士学位论文,成都中医药大学).

Pang, H. X. (2020). Clinical observation of “ Wrist-ankle Acupuncture with Filiform-fire needle” on Postherpetic neuralgia (Master Dissertation, Chengdu University of Traditional Chinese Medicine).

R24. 张海龙,尹洪娜,闫明,王俊志 & 刘刚.(2020).浮针联合“逆流补营”火针法治疗带状疱疹后遗神经痛临床研究. *针灸临床杂志*(11),38-41.

Zhang, H. L., Yin, H. N., Yan, M., Wang, J. Z., & Liu, G. (2020). Clinical research of Fu’s Subcutaneous Needling Combined with “Countercurrent Supplement of Ying” Fire Acupuncture in Treating PHN. Clinical Journal of Acupuncture and Moxibustion (11), 38-41.

R25. 徐先鹏.(2020).*基于“调神”理论针刺治疗带状疱疹后遗神经痛的临床观察*(硕士学位论文,黑龙江中医药大学).

Xu, X. P. (2020). Clinical observation on acupuncture treatment of postherpetic neuralgia based on the theory of “regulating the spirit” (Master Dissertation, Heilongjiang University of Chinese Medicine).

R26. 王丽,方玉甫,李庆娟,李静文 & 李伟玲.(2020).刺络拔罐对带状疱疹后遗神经痛患者血清P物质及IL-6的影响. 中国中医药现代远程教育(16),92-94.

Wang, L., Fang, Y. F., Li, Q. J., Li, J.W., & Li, W. L. (2020). Effect of Pricking and Cupping on Serum Substance P and IL-6 in Patients with Postherpetic Neuralgia. Chinese Modern Distance Education of Traditional Chinese Medicine (16), 92-94.

R27. 赵莹莹,许萍,毕军 & 张胜男.(2020).梅花针刺络拔罐治疗带状疱疹后遗神经痛患者的临床效果. *中国民康医学*(11),89-90+93.

Zhao, Y., Xu, P., Bi, J., & Zhang, S. N. (2020). Clinical effects of plum blossom needle acupuncture and cupping on patients with postherpetic neuralgia.

R28. 丁翔云,杨永明,丁影 & 张新.(2021).刺络拔罐联合加巴喷丁治疗中重度带状疱疹后遗神经痛患者的疗效及其对疼痛相关神经肽与炎性因子的影响. *中国中西医结合皮肤性病学杂志*(04),346-350.

Ding, X. Y., Yang, Y. M., Ding, Y., & Zhang, X. (2021). Efficacy of Bleeding and Cupping Combined with Gabapentin in the Treatment of Moderate to Severe Postherpetic Neuralgia Patients and Impact on Pain-related Neuropeptides and Inflammatory Cytokines. Chinese Journal of Dermatology and Venereology of Integrated Traditional and Western Medicine (04), 346-350.

R29. 张丽芹,丁锋 & 矫英.(2021).归芍颗粒、刺血拔罐法联合普瑞巴林对带状疱疹后遗神经痛患者的临床疗效. *中成药*(02),560-562.

Zhang, L. Q., Ding, F., & Jiao, Y. (2021). Clinical efficacy of pricking and cupping combined with pregabalin in patients with postherpetic neuralgia. Chinese Patent Medicine (02),560-562.
