# Supplementary figures and images for: Efficacy of different acupuncture therapies on postherpetic neuralgia: A Bayesian network meta-analysis
Source: Front Neurosci. 2023 Jan 10;16:1056102. doi: 10.3389/fnins.2022.1056102 (PMC9871906; doi:10.3389/fnins.2022.1056102)

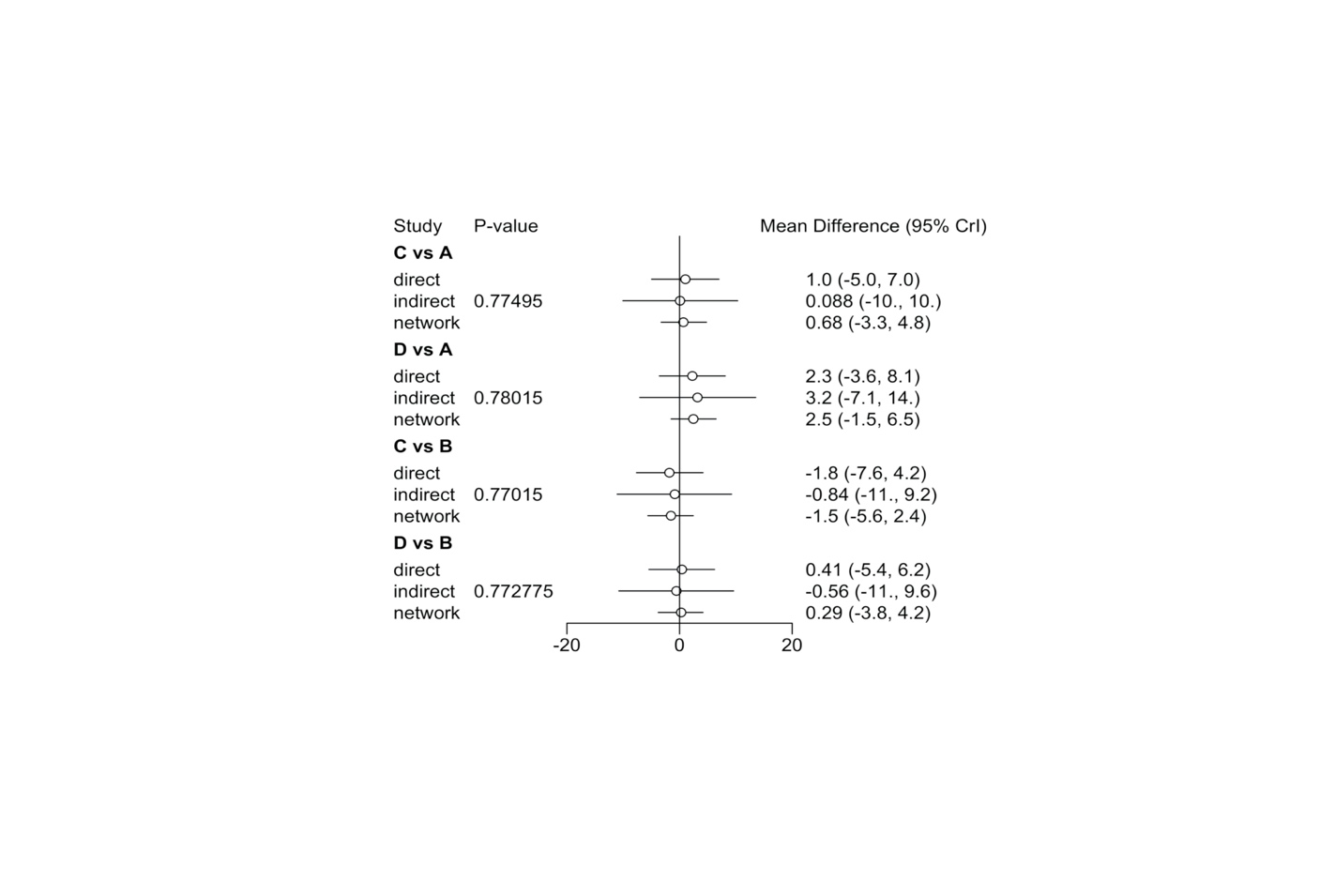

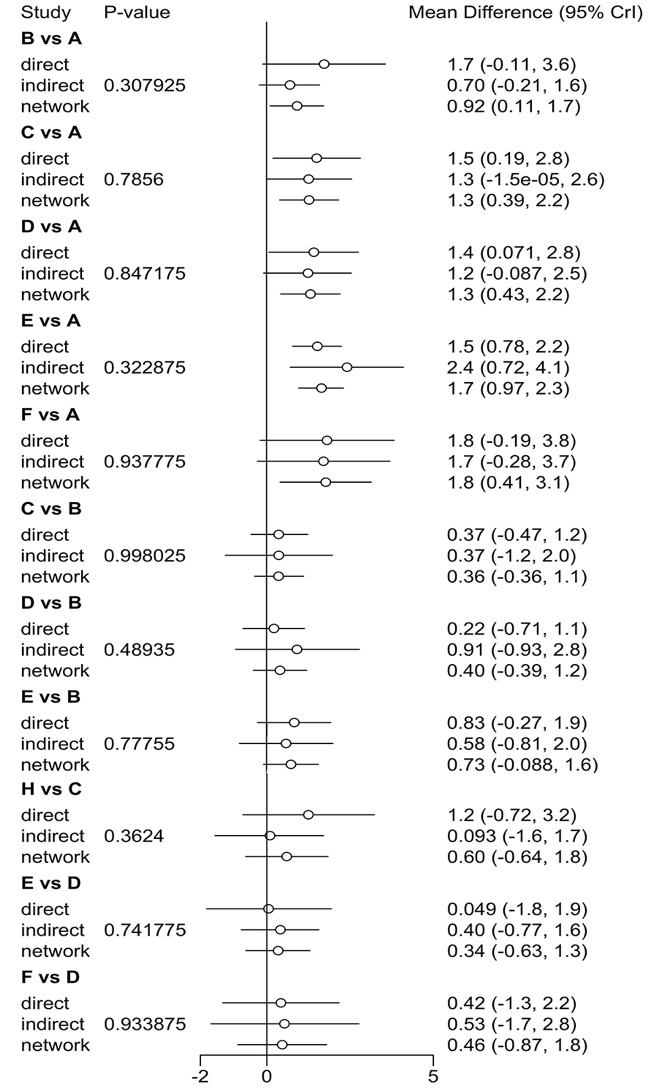

Supplement: Supplementary file 4 [file Data_Sheet_4.DOCX]
