# Supplementary material for: Efficacy of different acupuncture therapies on postherpetic neuralgia: A Bayesian network meta-analysis
Source: Front Neurosci. 2023 Jan 10;16:1056102. doi: 10.3389/fnins.2022.1056102 (PMC9871906; doi:10.3389/fnins.2022.1056102)
Supplement: Supplementary file 4 [file Data_Sheet_3.DOCX]

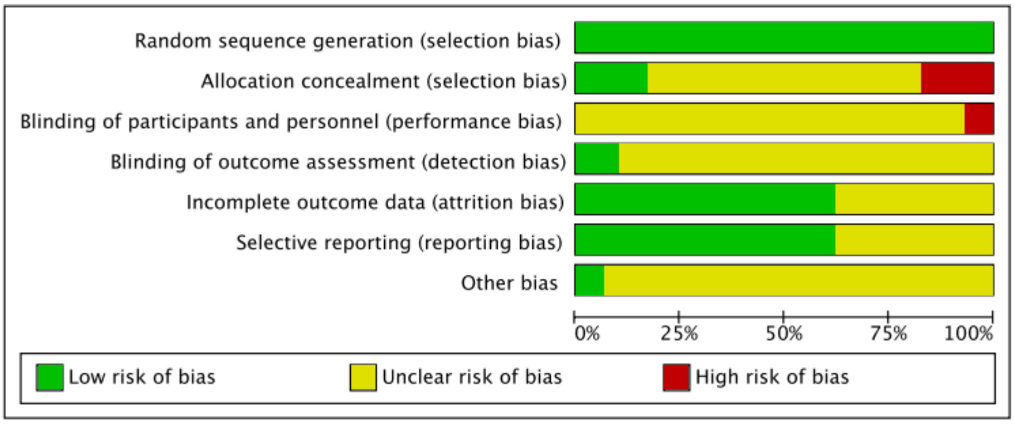
Random sequence generation (selection bias)
Allocation concealment (selection bias)
Blinding of participants and personnel (performance bias)
Blinding of outcome assessment (detection bias)
Incomplete outcome data (attrition bias)
Selective reporting (reporting bias)
Other bias
0%
25%
50%
75%
100%
Low risk of bias
Unclear risk of bias
High risk of bias

	Random sequence generation (selection bias)	Allocation concealment (selection bias)	Blinding of participants and personnel (performance bias)	Blinding of outcome assessment (detection bias)	Incomplete outcome data (attrition bias)	Selective reporting (reporting bias)	Other bias
Chen 2019	+	?	?	?	+	+	?
Chen et al. 2012	+	?	?	?	?	?	?
Chen et al. 2018	+	?	?	?	?	?	?
Ding et al. 2021	+	?	?	?	?	?	?
Duan 2014	+	?	?	?	+	+	?
Han et al. 2018	+	+	?	+	?	?	+
Hong 2016	+	?	?	?	+	+	?
Huang et al. 2011	+	?	?	?	?	?	?
Lei 2015	+	?	?	?	+	+	?
Mo 2019	+	+	?	?	+	+	?
Pang 2020	+	+	−	+	+	+	+
Sheng et al. 2014	+	?	?	?	?	?	?
Sun et al. 2015	+	?	?	?	+	+	?
Tian 2013	+	−	?	?	+	+	?
Wang et al. 2006	+	?	?	?	+	+	?
Wang et al. 2020	+	?	?	?	+	+	?
Wu et al. 2016	+	−	?	?	?	?	?
Xie et al. 2013	+	?	?	?	+	+	?
Xie et al. 2018	+	?	?	?	?	?	?
Xu 2018	+	?	?	?	+	+	?
Xu 2020	+	−	?	?	+	+	?
Yang et al. 2016	+	?	?	?	+	+	?
Zhang 2013	+	+	−	+	+	+	?
Zhang 2015	+	−	?	?	+	+	?
Zhang et al. 2020	+	−	?	?	+	+	?
Zhang et al. 2021	+	?	?	?	?	?	?
Zhao et al. 2020	+	?	?	?	?	?	?
Zhong 2019	+	+	?	?	+	+	?
Zhou et al. 2018	+	?	?	?	?	?	?
