# Supplementary material for: Efficacy of different acupuncture therapies on postherpetic neuralgia: A Bayesian network meta-analysis
Source: Front Neurosci. 2023 Jan 10;16:1056102. doi: 10.3389/fnins.2022.1056102 (PMC9871906; doi:10.3389/fnins.2022.1056102)
Supplement: Supplementary file 5 [file Data_Sheet_5.DOCX]

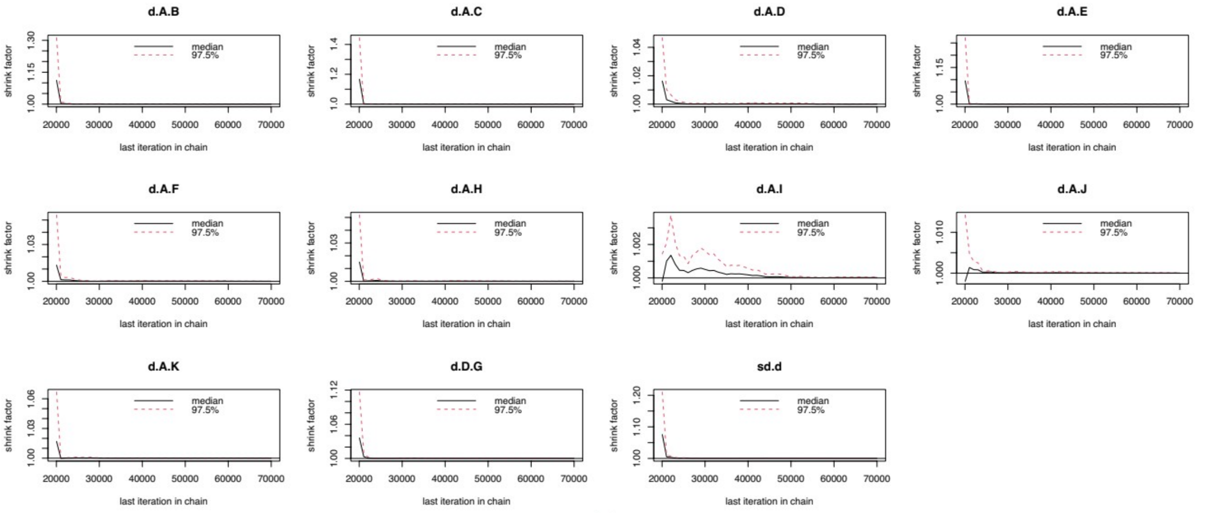

d.A.B
d.A.C
d.A.D
d.A.E
d.A.F
d.A.H
d.A.I
d.A.J
d.A.K
d.D.G
sd.d
median
97.5%
shrink factor
last iteration in chain
20000 30000 40000 50000 60000 70000

(a)

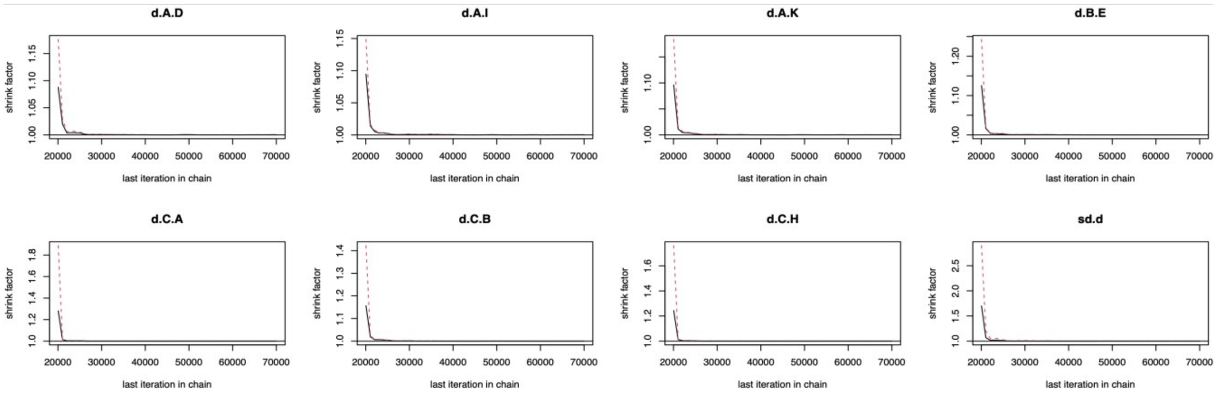

d.A.D
d.A.I
d.A.K
d.B.E
d.C.A
d.C.B
d.C.H
sd.d
shrink factor
last iteration in chain
20000 30000 40000 50000 60000 70000

(b)

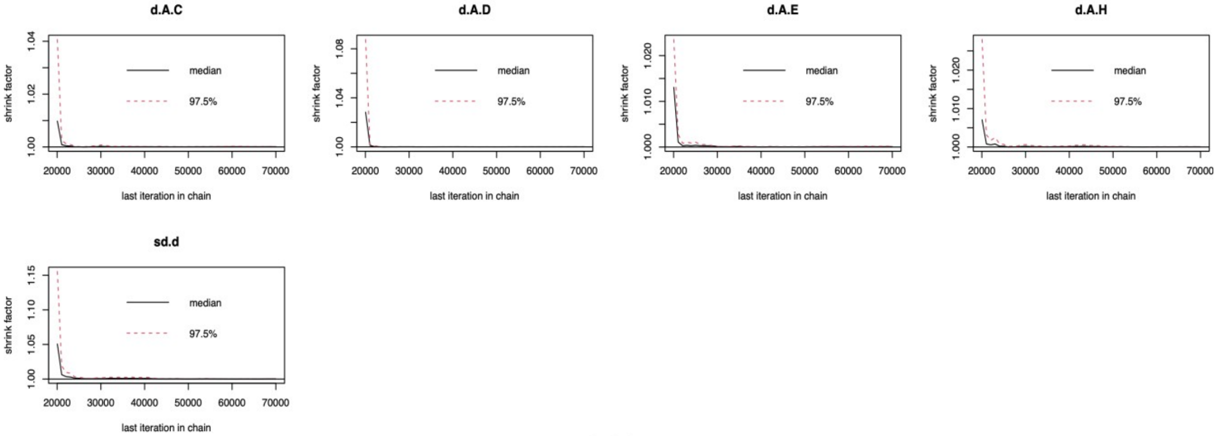

d.A.C
d.A.D
d.A.E
d.A.H
sd.d
median
97.5%
shrink factor
last iteration in chain
20000 30000 40000 50000 60000 70000

(c)
